# Orthology Analysis and *In Vivo* Complementation Studies to Elucidate the Role of DIR1 during Systemic Acquired Resistance in *Arabidopsis thaliana* and *Cucumis sativus*

**DOI:** 10.3389/fpls.2016.00566

**Published:** 2016-05-03

**Authors:** Marisa Isaacs, Philip Carella, Jennifer Faubert, Marc J. Champigny, Jocelyn K. C. Rose, Robin K. Cameron

**Affiliations:** ^1^Department of Biology, McMaster University, Hamilton, ON, Canada; ^2^Department of Molecular & Cellular Biology, University of Guelph, Guelph, ON, Canada; ^3^Plant Biology Section, School of Integrative Plant Science, Cornell University, Ithaca, NY, United States

**Keywords:** cucumber, DIR1, hydrophobic cavity, lipid transfer protein, long-distance signaling, systemic acquired resistance

## Abstract

AtDIR1 (Defective in Induced Resistance1) is an acidic lipid transfer protein essential for systemic acquired resistance (SAR) in *Arabidopsis thaliana*. Upon SAR induction, DIR1 moves from locally infected to distant uninfected leaves to activate defense priming; however, a molecular function for DIR1 has not been elucidated. Bioinformatic analysis and *in silico* homology modeling identified putative AtDIR1 orthologs in crop species, revealing conserved protein motifs within and outside of DIR1’s central hydrophobic cavity. *In vitro* assays to compare the capacity of recombinant AtDIR1 and targeted AtDIR1-variant proteins to bind the lipophilic probe TNS (6,*P*-toluidinylnaphthalene-2-sulfonate) provided evidence that conserved leucine 43 and aspartic acid 39 contribute to the size of the DIR1 hydrophobic cavity and possibly hydrophobic ligand binding. An *Arabidopsis*–cucumber SAR model was developed to investigate the conservation of DIR1 function in cucumber (*Cucumis sativus*), and we demonstrated that phloem exudates from SAR-induced cucumber rescued the SAR defect in the *Arabidopsis dir1-1* mutant. Additionally, an AtDIR1 antibody detected a protein of the same size as AtDIR1 in SAR-induced cucumber phloem exudates, providing evidence that DIR1 function during SAR is conserved in *Arabidopsis* and cucumber. *In vitro* TNS displacement assays demonstrated that recombinant AtDIR1 did not bind the SAR signals azelaic acid (AzA), glycerol-3-phosphate or pipecolic acid. However, recombinant CsDIR1 and CsDIR2 interacted weakly with AzA and pipecolic acid. Bioinformatic and functional analyses using the *Arabidopsis*–cucumber SAR model provide evidence that DIR1 orthologs exist in tobacco, tomato, cucumber, and soybean, and that DIR1-mediated SAR signaling is conserved in *Arabidopsis* and cucumber.

## Introduction

Systemic acquired resistance (SAR) is a plant defense response during which an initial infection leads to resistance to a broad spectrum of normally virulent pathogens in distant naive tissues. Pathogen-induced mobile SAR signals produced in locally infected leaves travel to distant leaves, resulting in signal perception and the manifestation of SAR. A number of physiological experiments demonstrated that mobile SAR signals travel from induced to distant tissues, predominantly via the phloem (reviewed in Guedes et al., 1980; Tuzun and Kuc, 1985; Champigny and Cameron, 2009). More recently this was also shown in *Arabidopsis* (Kiefer and Slusarenko, 2003). To date, a number of potential SAR mobile signals have been identified (reviewed in Dempsey and Klessig, 2012; Shah and Zeier, 2013; Shah et al., 2014), including lipid transfer proteins (LTPs; Maldonado et al., 2002; Jung et al., 2009; Xia et al., 2012; Champigny et al., 2013; Li et al., 2014; Cecchini et al., 2015), methyl salicylate (MeSA; Park et al., 2007; Vlot et al., 2008), azelaic acid (AzA; Jung et al., 2009; Wittek et al., 2014; Cecchini et al., 2015), a glycerol-3-phosphate (G3P)-derived molecule (Chanda et al., 2011), pipecolic acid (Pip; Navarova et al., 2012; Vogel-Adghough et al., 2013), and the abietane diterpenoid dehydroabietinal (DA; Chaturvedi et al., 2012). The existence of numerous putative SAR signals illustrates the complexity of the SAR signaling pathway and highlights the need to better understand the roles of these signals during SAR.

Since plants cannot predict which leaf will become infected, each leaf must have the capacity to produce SAR long-distance signals. Additionally, long-distance SAR signals must move from SAR-induced to distant leaves to establish SAR. The LTP DIR1 (Defective in Induced Resistance 1) possesses these characteristics as it is expressed in all living cells of leaves (Champigny et al., 2011) and experiments using an estrogen-inducible DIR1–GFP line provide compelling evidence that DIR1 is a mobile signal or chaperone that becomes activated in locally infected leaves to access the phloem and move to establish SAR in distant leaves (Champigny et al., 2013). Moreover, the resistance-promoting activity of G3P, AzA, and DA all require functional DIR1 (Jung et al., 2009; Chanda et al., 2011; Chaturvedi et al., 2012) and the SAR-related LTPs AzA Induced 1 (AZI1) and Early Arabidopsis Aluminum Induced 1 (EARLI1) have been shown to interact with DIR1 in transient expression experiments in *Nicotiana benthamiana* (Yu et al., 2013; Cecchini et al., 2015). These findings suggest that DIR1 participates as a member of a SAR signal complex. In support of this idea, a high molecular weight protein complex was identified in petiole exudates collected from SAR-induced leaves (Chaturvedi et al., 2012) and immunoblot analysis provided evidence that DIR1 is present in this complex (Shah et al., 2014). Taken together, these studies support the idea that DIR1 is an integral component of long-distance signaling during SAR.

Analysis of the DIR1 crystal structure revealed that DIR1 is a unique non-specific (ns)-LTP, most similar to members of the LTP2 family (Lascombe et al., 2008). Like other nsLTPs, DIR1 has eight cysteine residues that participate in four disulfide bonds to form a central hydrophobic cavity or pocket. Unlike other LTP2 proteins, DIR1 has an acidic isoelectric point (pI), it binds two monoacylated lipids within its hydrophobic pocket *in vitro* and it possesses a putative protein interaction PxxP motif (where P is proline and x is any amino acid; Lascombe et al., 2008). Given the characteristics of DIR1, it is possible that it interacts with lipids or other hydrophobic molecules, acting as a chaperone and/or as part of a larger protein complex that translocates from induced to distant tissues during SAR.

The importance of DIR1 in the SAR response is further supported by studies of DIR1 orthologs in other plant species. A putative DIR1 ortholog was identified in tomato and immunoblot analysis confirmed its presence in petiole exudates collected from healthy tomato plants (Mitton et al., 2009); however, its role during SAR was not investigated. Transgenic *Arabidopsis* plants expressing two putative DIR1 orthologs from *Nicotiana tabacum* rescued the SAR defect in the *Arabidopsis dir1-1* mutant and RNAi-mediated knockdown of these orthologs in *N. tabacum* impaired SAR (Liu et al., 2011). These studies suggest that DIR1 and DIR1-mediated SAR are conserved in other plants. Additionally, a DIR1-like protein with high sequence similarity to DIR1 (88% of the mature protein at the amino acid level) is present in *Arabidopsis*. Phylogenetic analysis, and the fact that *DIR1* and *DIR1-like* are adjacent to one another on chromosome 5, suggests they arose from a duplication event (Champigny et al., 2013). *DIR1* and *DIR1-like* are similarly expressed in naïve and pathogen-treated plants, and transiently expressed DIR1-like complements the *dir1-1* SAR defect (Champigny et al., 2013). Moreover, the *dir1-1* mutant occasionally displays a partially SAR-competent phenotype, suggesting that in some circumstances DIR1-like acts redundantly to DIR1 (Champigny et al., 2013).

To further understand the role of DIR1 during SAR, we used bioinformatic analyses and *in silico* homology modeling to identify and characterize orthologous DIR1 proteins from a number of agriculturally relevant plants. Conserved motifs in areas important for LTP structure were identified. Mutations were introduced into these motifs and their effect on the formation of the DIR1 hydrophobic cavity was examined. Further, we combined the cucumber and *Arabidopsis* SAR model systems to provide evidence that cucumber DIR1 orthologs are functionally equivalent to AtDIR1.

## Materials and Methods

### Plant Growth Conditions

Wild-type *Arabidopsis thaliana* Wassilewskija (Ws-2), *dir1-1*, and *npr1-2* seeds were surface sterilized and stratified at 4°C for 2 days in the dark. Sterilized seeds were plated on Murashige and Skoog (MS) plates and germinated under continuous light for 5–7 days. Seedlings were transplanted to soil hydrated with 1 g L^-1^ 20–20–20 fertilizer. *Arabidopsis* plants were grown in short day photoperiod conditions (9 h light; 150 μE m^-2^ s^-1^) in 65–85% relative humidity at 22°C. Cucumber Wisconsin S.M.R 58 146B seeds (Stokes Seeds LTD., St. Catharines, ON, Canada) were sown directly onto soil hydrated with 1 g L^-1^ 20–20–20 fertilizer and grown in 65–85% relative humidity at 22°C in a long day photoperiod (16 h light: 150 μE m^-2^ s^-1^).

### Pathogen Culture and Inoculation

Systemic acquired resistance experiments with *Arabidopsis* employed virulent *Pseudomonas syringae* pv. *tomato* (Pst) DC3000 (pVSP1) and avirulent *Pst* (DC3000 containing pVSP1 + avrRpt2) described in Whalen et al. (1991). SAR experiments with cucumber employed *P. syringae* pv. *syringae* D20 (Rasmussen et al., 1991). *Pseudomonas* strains were cultured overnight with shaking at room temperature in sterile King’s B (KB) medium (King et al., 1954). *Pst* cultures were supplemented with 100 μg ml^-1^ rifampicin and 50 μg ml^-1^ kanamycin. SAR assays in *Arabidopsis* were performed as described in Champigny et al. (2013). In cucumber, SAR was induced in 3–4 week-old plants by resuspending *Pss* D20 in 10 mM MgCl_2_ and infiltrating leaves with 10^8^ colony forming units (cfu) ml^-1^. *In planta* bacterial levels were quantified by dilution plating as described by Cameron et al. (1999).

### Cucumber Petiole Exudate Collection

Cucumber exudates were collected according to Rasmussen et al. (1991) by cutting the petiole on an angle 3–5 cm below the leaf blade with a razor blade. Exudate droplets (30–40 μl) were collected from the petiole cut ends using capillary pipettes and immediately added to 300 μl of cucumber exudate buffer (0.05 M Tris-HCl, pH 7.5 with 0.1% β-mercaptoethanol). Cucumber exudates contained between 5 and 15 μg μl^-1^ total protein (Biorad Protein Assay Kit). Samples were used immediately in cucumber–*Arabidopsis* SAR-rescue experiments or frozen at -20°C for later concentration by lyophilization and protein gel blot analysis.

### Agro-SAR and Petiole Exudate-Swapping SAR Assays

Agro-SAR assays were performed as described in Champigny et al. (2013) using *Agrobacterium tumefaciens* GV3101 (pMP90) expressing EYFP, DIR1-YFP, CsDIR1, or CsDIR2. The EYFP and DIR1-YFP constructs are described in Champigny et al. (2013). Cucumber DIR1 orthologs were cloned into pMDC32 (Curtis and Grossniklaus, 2003) to create the 35S:CsDIR1 and 35S:CsDIR2 constructs. Constructs were verified by sequencing. Cloning primers are described in Supplementary Table S1.

Cucumber petiole exudates were collected from leaves that were mock-inoculated or induced for SAR with 10^7^ cfu ml^-1^ *Pseudomonas syringae* pv *syringae* D20 at 8 and 22 h post inoculation (hpi). Cucumber exudates were filter sterilized (0.45 μm, EMD Millipore) and samples were stored at -20°C for future lyophilization and protein gel blot analysis or used immediately in the cucumber–*Arabidopsis* SAR assay. Cucumber exudates from mock-inoculated and SAR-induced leaves were diluted 10-fold in sterile distilled water then pressure-infiltrated into two lower *Arabidopsis* leaves using a needleless syringe. Two days later, distant upper leaves were challenge-inoculated with virulent *Pst* at 10^5^ cfu ml^-1^, followed by *in planta Pst* quantitation 3 days post-inoculation (dpi).

### Phylogenetic and Bioinformatic Analyses

A rooted phylogenetic Maximum Likelihood tree was created for AtDIR1 (AT5G48485), Brassicaceae ortholog family members and crop plant DIR1 orthologs. The phylogeny used protein sequences lacking the divergent ER signal sequence. Signal P 4.0 was used to determine the location of the signal sequence cleavage site (Perterson et al., 2011). The sequences were aligned in MEGA 5 using Muscle (Tamura et al., 2011). The evolutionary history was inferred using the Maximum Likelihood method based on the Kimura 2-parameter (Kimura, 1980) model with discrete Gamma distribution using MEGA 5 (Tamura et al., 2011). A total of 10,000 bootstrap replicates were conducted and percent bootstrap values were placed on the branches (Felsenstein, 1985). Branches were drawn to scale, measured in number of substitutions per site and were labeled by species name followed by The Arabidopsis Information Resource (TAIR^1^) gene number or Phytozome 8.0 accession (Goodstein et al., 2012). For the Brassicaceae plus crop plant DIR1 ortholog phylogeny, branches with <50% bootstrap values were collapsed using Archaeopteryx software (Han and Zmasek, 2009). The phylogenetic tree was viewed in FigTree v1.4 (Drummond et al., 2012).

The coding sequences and amino acid sequences of AtDIR1 (AT5G48485) and AtDIR1-like (AT5G48490), AtLTP2.12 (AT5G38170), were retrieved from TAIR. Tobacco DIR1 ortholog sequences were retrieved from the National Center for Biotechnology Information (NCBI^2^) website. Cucumber, tomato, and soybean DIR1 ortholog sequences were retrieved using Phytozome^3^. Sequences were compared using the EMBOSS Needleman–Wunsch pairwise alignment algorithm^4^. Signal peptides were deduced using the SignalP 3.0 prediction server^5^ (Perterson et al., 2011). SWISS-MODEL homology models of AtDIR1-like, CsDIR1 and CsDIR2 were produced using the AtDIR1-phospholipid crystal structure (Lascombe et al., 2008) as a template (Peitsch, 1995; Guex and Peitsch, 1997; Schwede et al., 2003; Arnold et al., 2006; Kiefer et al., 2009). The Swiss-pdf viewer 4.0.1 and ICM browser were used to compare the AtDIR1 structure and the AtDIR1-like, CsDIR1, and CsDIR2 protein models^6^ (Guex and Peitsch, 1997). A Sequence Logo plot was created by submitting the Muscle aligned mature DIR1 ortholog protein sequences in FASTA format to the Web logo program^7^.

### DIR1 Variant Cloning and Recombinant Protein Purification

DIR1 variants were synthesized by BioBasic Inc (Markham, ON, Canada). Full sequence information for each variant can be found in Supplementary Document 1. Variants lacking the N-terminal ER signal sequence were subcloned into the pET29b expression vector (Novagen) using the primers in Supplementary Table S1 and were verified by sequencing. The constructs were transformed into competent Rosetta Gami *E. coli* cells (Novagen). For protein expression, 250 and 500 ml Rosetta Gami *E. coli* cultures were grown overnight in liquid LB (Luria–Bertani) at 30°C with shaking at 200 rpm. At an OD_600_ of 0.6, 100 ml of each culture was poured into a new flask and 1 mM isopropyl β-D-1-thiogalactopyranoside (IPTG, BioShop) was added to induce protein expression. Cultures were shaken for another 4 h at 30°C. Cells were harvested by centrifugation in two sterile 50 ml tubes at 4000 × *g* for 20 min at 4°C. One milliliter of cells was also collected for crude protein extraction. The pellets were dried and kept -80°C prior to either a crude total protein extraction or S-Tag thrombin protein purification (Novagen).

For crude extraction of total protein, pellets from 1 mL of IPTG-induced *E. coli* cells were resuspended in 100 μl of lysis buffer (140 mM NaCl, 2.7 mM KCl, 1.8 mM KH_2_PO_4_, Phosphate Buffered Saline pH 7.3, 1 mM phenylmethylsulfonyl fluoride, 0.1% TritonX-100). This mixture was sonicated three times for 10 s each (60% amplitude, cooling on ice in between. After lysis the cells were centrifuged for 5 min at 20,000 × *g* (4°C) to separate soluble and insoluble fractions.

An S-Tag thrombin purification kit (Novagen) was used to purify the recombinant Rosetta Gami expressed proteins. Proteins were purified according to manufacturer’s instructions (Novagen) and quantified (Biorad Protein Assay Kit) using bovine serum albumin (BSA) as a standard. Samples were stored at -80°C until further use.

### Immunoblot Analysis

Protein samples (crude protein extract, purified protein or petiole exudates concentrated by lyophilization) were mixed with 5x SDS loading buffer (350 mM Tris-HCl pH 6.8, 30% glycerol, 10% SDS, 0.01% bromophenol blue and 200 mM dithiothreitol) followed by boiling for 5 min. Samples were loaded onto 4–12% NuPAGE Bis-Tris polyacrylamide gels (Life Science Technologies) and subjected to electrophoresis in denaturing MES running buffer (9.7 g L^-1^ MES, 6.0 g L^-1^ Tris, 1 g L^-1^ SDS, 0.37 g L^-1^ EDTA). Proteins were transferred to nitrocellulose membranes in Towbin transfer buffer (25 mM Tris base, 192 mM glycine, 20% methanol, pH 8.3). Membranes were probed with anti-DIR1 antisera (Maldonado et al., 2002) at a 1:20,000 dilution, or anti-6-His (Covance) at a 1:3,000 dilution in 5% non-fat milk in 1X Tris Buffered Saline with Tween 20 (50 mM Tris-HCl pH 7.5, 150 mM NaCl, 0.5% Tween 20). Antibody binding was detected with a goat anti-rabbit (AtDIR1-antibody) or goat anti-mouse (His-antibody, Sigma–Aldrich) horseradish peroxidase conjugate and WestFemto reagents (Pierce) as described by the manufacturer.

### TNS Binding Assay and TNS Displacement Experiments

Proteins were engineered to resemble mature protein lacking the ER signal sequence and were expressed in Rosetta Gami *E. coli*. For TNS binding assays, increasing concentrations (0–30 μM) of 6,*P*-toluidinylnaphthalene-2-sulfonate (TNS, Sigma–Aldrich) were added to 1 μg of each Rosetta Gami *E. coli* (Novagen) purified protein in measurement buffer (0.5 mM K_2_SO_4_, 0.5 mM CaCl_2_, 0.175 M mannitol, 5 mM MES, pH 7) and TNS concentrations were increased in 5 μM increments from a 0.3 mM TNS stock (dissolved in DMSO). As a control, proteins were denatured by boiling in a solution of 6 M urea for 10 min and analyzed. Samples were loaded into microflor two black bottom 96 well microtiter plates (Thermo Fisher) and analyzed using the Gen5 Synergy 4 plate reader (BioTek Instruments). Experiments using the DIR1^ΔCys^ variant were performed using an Infinite M1000 (TECAN) plate reader. TNS-binding curves displayed similar trends irrespective of the instrument used. Samples were excited at 320 nm and emission was collected at 437 nm. The change in fluorescence (*x* μM TNS – 0 μM TNS) was calculated for three technical replicates.

6,*P*-Toluidinylnaphthalene-2-sulfonate displacement/ligand binding assays were performed using the same plates and instrument. Fluorescence was first measured when TNS (5 μm) and putative ligands (16 μm AzA, pipecolic acid, or G3P; Sigma–Aldrich) were co-incubated for 3 min in a 96 well plate, then a second reading was recorded after purified proteins were added. Plates were loaded with 1 μg of protein in measurement buffer (0.5 mM K_2_SO_4_, 0.5 mM CaCl_2_, 0.175 M mannitol, 5 mM MES, pH 7) and 5 μM TNS. AzA was prepared in 5 mM MES (pH 5.6), while all other putative ligands were prepared in water. Fluorescence was measured at an excitation wavelength of 320 nm and emission was collected at 437 nm in technical triplicate. Ligand binding was represented by the percent of TNS fluorescence that was quenched by the addition of the purified proteins.

### RNA Isolation and Reverse Transcription (RT)-PCR

Total RNA was isolated from frozen leaf tissue using the Sigma TRI-Reagent as previously described (Carella et al., 2015). Primers for RT-PCR analysis can be found in Supplementary Table S1. Twenty-eight PCR cycles were used.

## Results

### Identification of Putative DIR1 Orthologs

**FIGURE 1:**
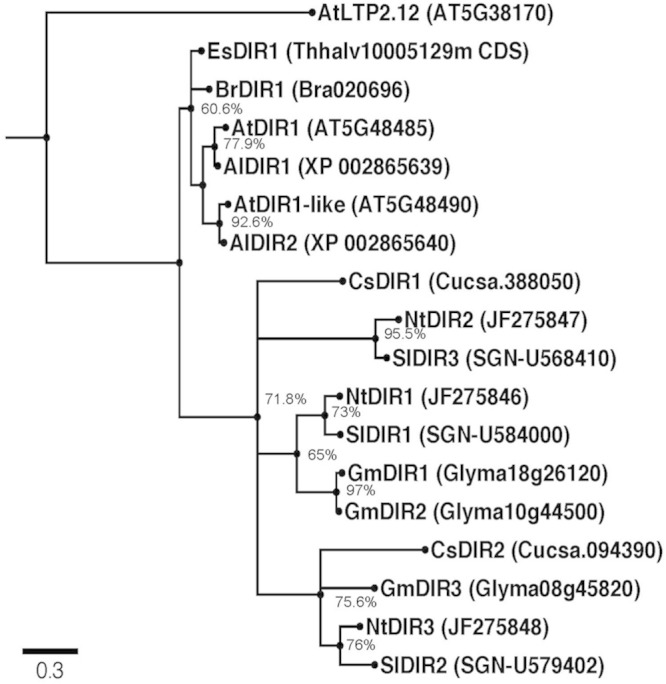
**Rooted Phylogenetic Maximum Likelihood tree of putative DIR1 orthologs.** DNA sequences lacking the divergent ER signal sequence were aligned using MUSCLE. The evolutionary history was inferred using the Maximum Likelihood method based on the Kimura 2-parameter model with discrete Gamma distribution using MEGA 5. Ten thousand bootstrap replicates were conducted and percent bootstrap values were placed on corresponding branches. Nodes with bootstrap values below 50% were collapsed using Archaeopteryx software. Phylogeny was viewed in FigTree v1.4. Branches were drawn to scale, measured in number of substitutions per site and branches were labeled by species name followed by TAIR gene number or Phytozome 8.0 accession. Al: *Arabidopsis lyrata*, At: *Arabidopsis thaliana*, Br: *Brassica rapa*, Cs: *Cucumis sativus*, Es: *Eutrema salsugineum*, Gm: *Glycine max*, Nt: *Nicotiana tabacum*, Sl: *Solanum lycopersicum.*

To gain insight into the phylogenetic relationships among DIR1 orthologs, we identified and examined putative DIR1 sequences in model and crop plants. Putative DIR1 orthologs from members of the Brassica family (*Arabidopsis thaliana, Arabidopsis lyrata, Brassica rapa, Eutrema salsugineum*), *Nicotiana tabacum* (tobacco), *Solanum lycopersicum* (tomato), *Cucumis sativus* (cucumber), and *Glycine max* (soybean) were identified as having >51% amino acid sequence similarity with mature AtDIR1 (**Table 1**). The evolutionary relationships between AtDIR1 and putative orthologs were examined by constructing a phylogenetic tree (**Figure 1**). We selected the *Arabidopsis* ortholog of wheat LTP2, AtLTP2.12 (At5g38170; Edstam et al., 2011), as an outgroup because it has only 38% amino acid sequence similarity to AtDIR1. As with the *DIR1 Brassica* phylogeny described previously (Champigny et al., 2013), this expanded phylogeny (**Figure 1**) predicted that AtDIR1 and AtDIR1-like were the result of a tandem duplication event in an ancestor of *A. lyrata* and *A. thaliana.* Independent DIR1 duplications in tomato, cucumber, tobacco, and soybean are also predicted. A lineage-specific duplication event was predicted in soybean, resulting in *GmDIR1* and *GmDIR2*, while duplication events in cucumber, tomato, and tobacco occurred in a common ancestor. Moreover, the *Arabidopsis DIR1/DIR1-like* and cucumber *DIR1/DIR2* duplications occurred independently. This DIR1 phylogeny provided evidence that DIR1 is conserved in agriculturally relevant plant species. Additionally, the identification of several DIR1 orthologs facilitates the discovery of conserved motifs and residues that can be used to learn more about DIR1 structure and function.

**Table 1:** Amino acid sequence similarity and identity of putative DIR1 orthologs.

Organism	Protein	Amino Acid Sequence	
		Similarity^a^	Identity^a^
*Arabidopsis thaliana*	AtDIR1-like	88%	80%
	AtLTP2.12^b^	38%	27%
*Arabidopsis lyrata*	AlDIR1	96%	93%
	AlDIR1-like	87%	80%
*Brassica rapa*	BrDIR1	83%	72%
*Eutrema salsugineum*	EsDIR1	86%	77%
*Nicotiana tabacum*	NtDIR1	62%	52%
	NtDIR2	57%	44%
	NtDIR3	67%	51%
*Solanum lycopersicum*	SlDIR1	63%	50%
	SlDIR2	63%	47%
	SlDIR3	58%	46%
*Cucumis sativus*	CsDIR1	61%	48%
	CsDIR2	65%	47%
*Glycine max*	GmDIR1	59%	47%
	GmDIR2	57%	46%
	GmDIR3	61%	49%

*^a^Mature protein lacking signal peptide. ^b^Outgroup for phylogenetic analysis.*

### Homology Modeling Supports the Existence of DIR1 Orthologs in *Cucumis sativus*

**FIGURE 2:**
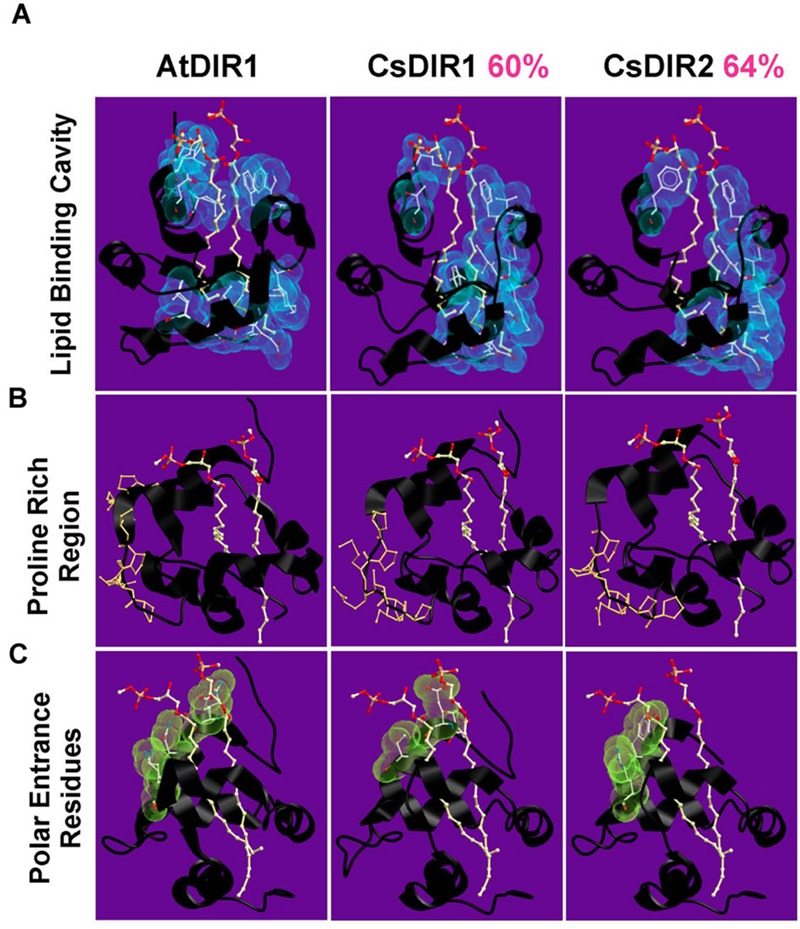
**Homology models of CsDIR1 and CsDIR2.** Proteins were modeled with SWISS-MODEL 4.0.1 server using AtDIR1-phospholipid crystal structure as a template and modeled with SWISS-MODEL 4.0.1 server and viewed using Molsoft ICM browser. Percent sequence similarity compared to AtDIR1 is identified in pink. **(A–C)** phospholipids in orange (phosphate), red (oxygen), and white (carbon). **(A)** The hydrophobic residues (blue) that are within 5 Å of the phospholipids highlight the inner hydrophobic cavity. **(B)** The proline residues of the proline rich regions are highlighted in orange. **(C)** The polar residues at the cavity entrance are highlighted in green.

The cucumber SAR model system was used in seminal SAR studies to investigate the nature of long-distance signaling (Guedes et al., 1980; Rasmussen et al., 1991; Smith-Becker et al., 1998), largely due to the ability to directly collect phloem sap from cut cucumber petioles. Therefore, we chose to investigate DIR1 orthologs in this well-developed SAR model system. Homology modeling was used to compare the protein structure of AtDIR1 and the putative cucumber DIR1 orthologs CsDIR1 and CsDIR2. Using the AtDIR1-phospholipid crystal structure (Lascombe et al., 2006, 2008) as a template, a homology model of each cucumber ortholog was generated using the SWISS-MODEL server (**Figure 2**) (Peitsch, 1995; Guex and Peitsch, 1997; Schwede et al., 2003; Arnold et al., 2006; Kiefer et al., 2009). AtDIR1, CsDIR1, and CsDIR2 all share a similar hydrophobic cavity into which hydrophobic phospholipid tails may extend (**Figure 2A**). The AtDIR1 PxxPxxP motif is a proposed protein–protein interaction site (Lascombe et al., 2008). CsDIR1 (PxPxxxPP) and CsDIR2 (PPxPxPP) contain a proline-rich region, rather than the canonical PxxPxxP motif (**Figure 2B**). Another region considered to be important for *in vitro* phospholipid docking is the entrance of the hydrophobic cavity, which contains hydrophilic residues. Lascombe et al. (2008) postulated that these charged residues interact with the hydrophilic region of putative lipid head groups, while the hydrophobic acyl tails are bound within the hydrophobic cavity. AtDIR1 possesses three hydrophilic residues within 5 Å of the phospholipid head groups (GLN9, ASN13, LYS16), while CsDIR1 and CsDIR2 both possess two residues (TYR7, ARG10 and GLU8, THR12, respectively; **Figure 2C**). Homology models of the putative DIR1 orthologs from tobacco, tomato, and soybean were generated and all were structurally similar to AtDIR1 (data not shown). Homology modeling of AtDIR1 and the putative DIR1 orthologs identified common structural motifs, providing additional support for the importance of these motifs for DIR1 function. Moreover, the high degree of structural similarity between cucumber, tobacco, and soybean DIR1 proteins with AtDIR1 validates our bioinformatics analyses and provides support for the list of putative DIR1 orthologs (**Table 1**; **Figure 1**).

### Sequence Alignment of DIR1 Orthologs Identifies Conserved Motifs

**FIGURE 3:**
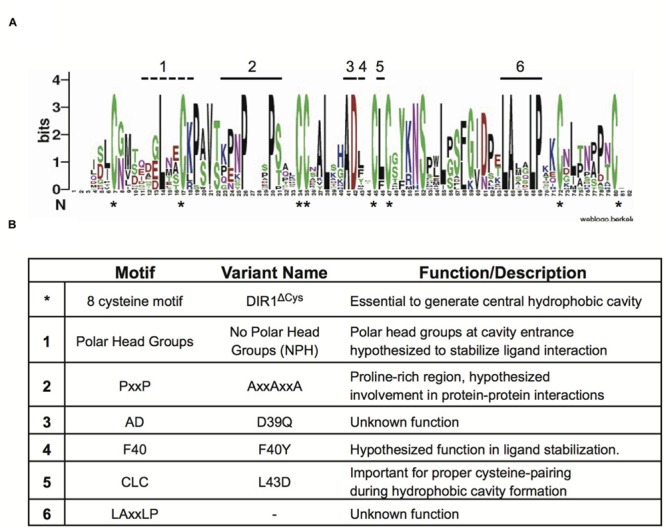
**Identification of conserved amino acid residues and motifs among DIR1 orthologs. (A)** Sequence Logo plot of Muscle aligned mature DIR1 orthologs protein sequences. Residues of the orthologs are stacked on top of one another and their frequency determines the height of the residue letter in the plot. Bits is a measure of the information content at that position in the sequence. Conserved cysteine residues are indicated by an asterisk (^∗^) that appears under the *x*-axis. Other conserved areas are highlighted with numbers above the corresponding letter. **(B)** Summary of identified motifs. Motif name, DIR1 variant protein generated, and a short description are included.

Given that the DIR1 phylogeny presented in **Figure 1** supports the existence of DIR1 orthologs, we used these DIR1 sequences to identify additional conserved protein motifs that may be important for DIR1 function during SAR. To identify conserved protein motifs a Sequence Logo was created using the online Web Logo algorithm (Schneider and Stephens, 1990; Crooks et al., 2004). This provides a visual output of multiple sequence alignments, where residues are stacked on top of one another and their frequency determines the height of the residue letter, allowing identification of conserved regions. Alignment of AtDIR1 and predicted DIR1 orthologs (**Table 1**) was performed using MEGA and submitted to the Sequence Logo online application. The Sequence Logo output (**Figure 3A**) predicted several conserved regions (**Figure 3B**), including the eight-cysteine motif that forms the four intramolecular disulfide bonds essential for LTP protein structure. The proline rich PxxP-like regions, which represent possible sites of protein–protein interaction, were common to all DIR1 proteins. Moreover, two previously unidentified motifs, AD and LAxxLP, were identified. Both motifs are located at the bottom of the hydrophobic cavity. In the AD motif, alanine faces inward toward the hydrophobic cavity and aspartic acid reaches outward toward the solution, while the LAxxLP motif is exposed to the solution. The polar tyrosine residue inside the hydrophobic cavity of AtDIR1-like (Y40 of mature polypeptide) was only present in AtDIR1-like and its corresponding putative ortholog in *A. lyrata*, while the predicted DIR1 orthologs had various non-polar residues at this location.

### Modification of AtDIR1 to Investigate Conserved Motif Function

The importance of the motifs identified in the Sequence Logo was investigated by creating several DIR1 variants with amino acid substitutions in these regions (**Figure 3B**). The PxxPxxP motif in AtDIR1 was selected for modification because of its high conservation among putative DIR1 orthologs and because it could be a site of protein–protein interactions (Lascombe et al., 2008). Multiple PxxP motifs are thought to strengthen such interactions (Williamson, 1994), therefore each proline residue was changed to an alanine (PxxPxxP to AxxAxxA). Although not identified in the Sequence Logo alignment, the polar amino acids at the DIR1 hydrophobic cavity entrance (Gln9, Asn13, Lys16) may be essential for stabilizing ligand binding (Lascombe et al., 2008). These residues were investigated using a variant in which the polar head group residues are changed to alanine to create the “No Polar Head group” (NPH) DIR1 variant (Gln9Ala, Asn13Ala, Lys16Ala).

The hydrophobic pocket of DIR1 was investigated using a DIR1 variant in which non-polar phenylalanine 40 is modified to tyrosine to resemble AtDIR1-like, which has a polar tyrosine at this position. This difference is hypothesized to affect the ability of AtDIR1-like to participate in SAR by disrupting the integrity of the pocket and/or reducing ligand interactions (Champigny et al., 2013). This idea is supported by the Sequence Logo alignment in which only AtDIR1-like and AlDIR1-like possessed a polar residue at this location. Leucine 43 lies between two cysteine residues in the hydrophobic cavity of many nsLTP2 proteins and has been shown to be important for protein folding, therefore a DIR1 variant with Cys-Asp43-Cys (L43D) was created (Samuel et al., 2002; Cheng et al., 2008). As an additional control, a DIR1 variant that lacks all 8 of the conserved cysteine residues required for disulfide bond formation was created (DIR1^ΔCys^, eight alanine residues substituted for eight cysteine residues) to generate an unfolded protein lacking the hydrophobic pocket entirely.

### *In Vitro* TNS Binding Assays to Compare Hydrophobic Cavities of Recombinant DIR1 and DIR1 Variant Proteins

**FIGURE 4:**
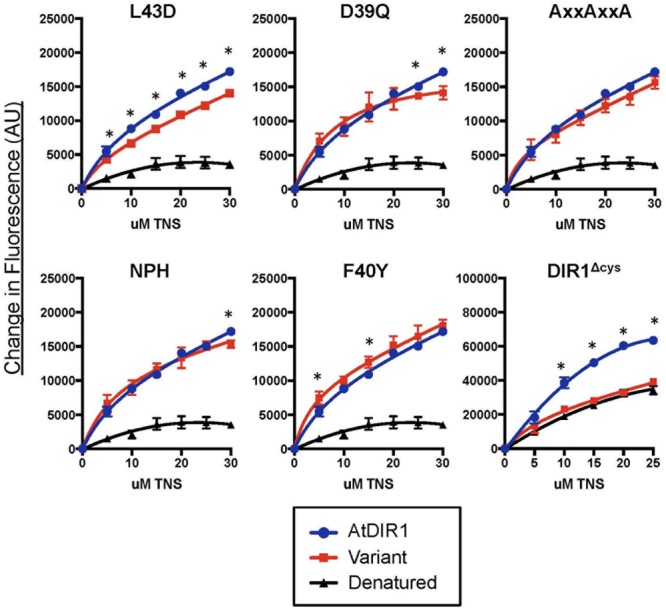
**Comparative *in vitro* TNS (6,*P*-toluidinylnaphthalene-2-sulfonate) binding assays of recombinant DIR1 variant proteins and AtDIR1.** DIR1 variants (L43D, D39Q, AxxAxxA, NPH, F40Y, and DIR1^Δcys^: red lines, squares) are compared to natured (blue lines, circles) and denatured (black lines, triangles) AtDIR1 protein. Increasing concentrations of TNS were added to each Rosetta Gami *E. coli* purified protein lacking the ER signal sequence. TNS binding curves were generated in PRISM6 by non-linear curve fitting for one site saturation binding. Proteins were denatured by boiling in 6 M Urea. Samples were excited at 320 nm and emission at 437 nm and the change in fluorescence was calculated for three replicates. Error bars represent the standard deviation. Significant differences between AtDIR1 and each variant at a given concentration of TNS were determined by Student’s *t*-test (*p* < 0.05) and are indicated by an asterisk (^∗^). The DIR1^Δcys^ data was obtained using a different instrument (see Section “Materials and methods”).

**FIGURE 5:**
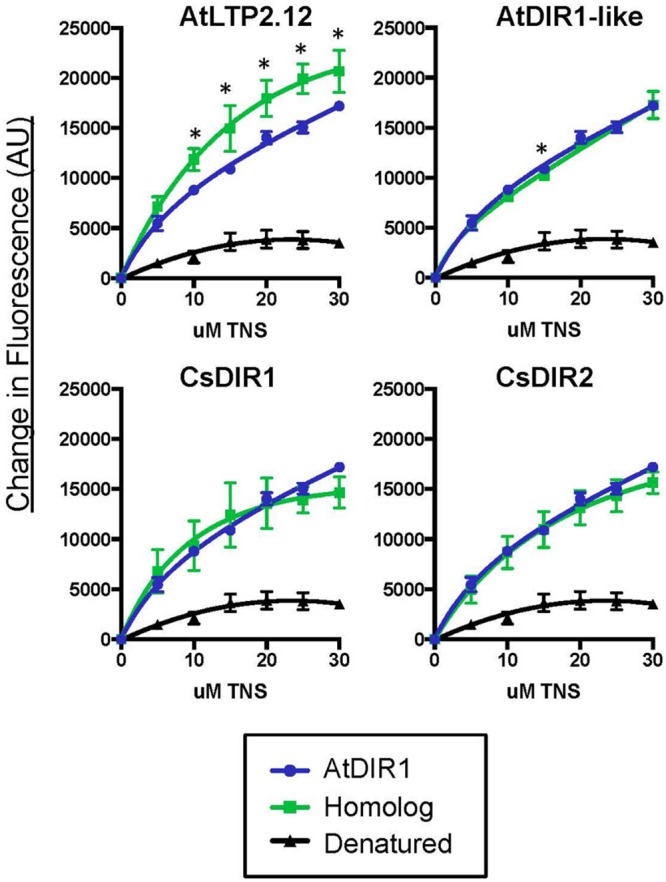
**Comparative *in vitro* TNS binding assays of recombinant AtDIR1, AtDIR1-like, AtLTP2.12, and the CsDIR1/CsDIR2 orthologs.** DIR1 homologs and the AtLTP2.12 control (green lines, squares) are compared to natured (blue lines, circles) and denatured (black lines, triangles) AtDIR1 protein. Increasing concentrations of TNS were added to each Rosetta Gami *E. coli* purified protein lacking the ER signal sequence. TNS binding curves were generated in PRISM6 by non-linear curve fitting for one site saturation binding. Proteins were denatured by boiling in 6 M Urea. Samples were excited at 320 nm and emission at 437 nm and the change in fluorescence was calculated for three replicates. Error bars represent the standard deviation of the technical variation. Significant differences between AtDIR1 and each homolog at a given concentration of TNS were determined by Student’s *t*-test (*p* < 0.05) and are indicated by an asterisk (^∗^).

Recombinant protein of AtDIR1, AtDIR1-like, AtLTP2.12, CsDIR1, CsDIR2, and the DIR1 variants discussed above were expressed in Rosetta Gami *E. coli*. Unlike many *E. coli* strains used to isolate recombinant proteins, Rosetta Gami *E. coli* promotes disulfide bond formation of proteins in the bacterial cytosol (Rosano and Ceccarelli, 2014). Disulfide bond formation contributes to formation of the LTP hydrophobic cavity (Desormeaux et al., 1992; Douliez et al., 2000; Yeats and Rose, 2008). To confirm that the proteins produced in Rosetta Gami *E. coli* were folded, and to begin to investigate the structural effects of modifying the conserved DIR1 motifs identified above (**Figure 3**), a protein-folding assay, based on the lipophilic synthetic probe TNS, was employed. TNS fluoresces in hydrophobic environments (McClure and Edelman, 1966) and is routinely used to investigate the hydrophobic cavities of LTPs (Mikes et al., 1998; Girault et al., 2008; DeBono et al., 2009; Guo et al., 2013). The formation of a hydrophobic cavity was examined by incubating each Rosetta Gami-generated recombinant protein with increasing concentrations of TNS and measuring the change in fluorescence. As a control, TNS binding curves of recombinant protein were compared to curves generated using protein samples that were denatured by boiling in urea. The TNS binding curves of each DIR1 variant is shown alongside folded and denatured AtDIR1 to illustrate the TNS binding capacity of each protein relative to AtDIR1 (**Figure 4**). Individual TNS binding curves for each protein (folded and denatured) are shown in **Supplementary Figure S1** and batch-to-batch repeatability of TNS assays for selected proteins is shown in **Supplementary Figure S2**. Denatured recombinant proteins displayed little change in fluorescence with increasing TNS, and were used as negative controls for TNS binding (**Figures 4** and **5**; **Supplementary Figures S1** and **S2**). In assays containing native AtDIR1 and DIR1 variant proteins, fluorescence increased with increasing TNS concentrations in comparison to denatured protein negative controls, providing evidence that Rosetta Gami *E. coli* produced properly folded LTP proteins (**Figure 4**). The AxxAxxA, F40Y, and NPH variants displayed statistically similar TNS binding profiles in comparison to AtDIR1, which suggests that these residues are not essential for the formation or maintenance of the hydrophobic cavity. As expected, the TNS binding curve of the DIR1^ΔCys^ variant was similar to that of denatured AtDIR1, confirming the importance of the disulfide bonds of DIR1 for protein folding. Interestingly, TNS fluorescence was reduced in assays containing the L43D and D39Q variants compared to native AtDIR1 (**Figure 5**), indicating that replacing these hydrophobic cavity residues with charged or polar residues reduced the TNS-DIR1 interaction.

### TNS Displacement Assays to Determine whether *Arabidopsis* or Cucumber DIR1 Proteins Interact with Putative SAR Signals

Fluorescence displacement assays can be used to assess *in vitro* binding of lipids to LTP proteins (Buhot et al., 2004; Krasikov et al., 2011; Lei et al., 2014). To determine if AtDIR1 and/or DIR1 homologs from *Arabidopsis* or cucumber interact with known SAR-activating small molecules, we purified recombinant AtDIR1, AtDIR1-like, CsDIR1, CsDIR2, and the unrelated AtLTP2.12 protein from Rosetta Gami *E. coli* for use in fluorescence displacement assays. Before performing ligand-interaction assays, the folding status of each recombinant protein was assessed using *in vitro* TNS binding assays. In assays comparing native AtDIR1, AtDIR1-like, CsDIR1, CsDIR2, and AtLTP2.12, fluorescence increased with increasing TNS concentrations in comparison to denatured protein controls, providing evidence that these LTP proteins are properly folded (**Figure 5**). Interestingly, the unrelated AtLTP2.12 protein displayed significantly higher TNS fluorescence compared to AtDIR1 with 10, 20, and 30 μM TNS (Student’s *t*-test, *p* < 0.05). This result was expected, as AtLTP2.12 is a putative ortholog of wheat LTP2 that has two hydrophobic cavities with volumes of 300 and 103 Å^3^ (Hoh et al., 2005) compared to AtDIR1, which has a single hydrophobic cavity with a volume of 242 Å^3^ (Lascombe et al., 2008).

**FIGURE 6:**
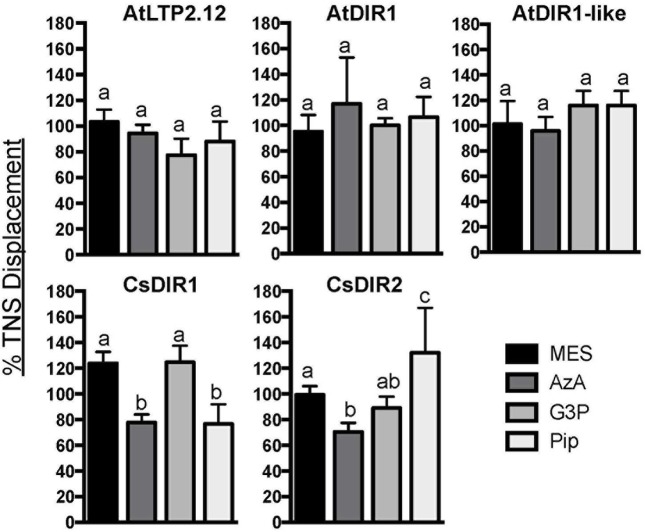
***In vitro* ligand binding assays.** TNS displacement assays using AtDIR1, AtLTP2.12, AtDIR1-like, CsDIR1, and CsDIR2 to test the binding of azelaic acid (AzA), glycerol-3-phosphate (G3P), pipecolic acid (Pip), and MES buffer control. Fluorescence was measured when TNS (3 μm) and putative ligands (16 μm) were incubated together for 3 min, then again after purified proteins were added. Fluorescence was measured at excitation wavelength of 320 nm and emission at 437 nm in triplicate. The data is represented as the percent TNS Displacement, which indicates the fluorescence level of protein-ligand-TNS compared to TNS-protein alone (100%). Error bars indicate standard deviation of three replicate measurements. Different letters indicate significant differences (ANOVA, Tukey’s HSD, *p* < 0.05).

To investigate whether the AtDIR1 hydrophobic cavity interacts with known SAR signal molecules, TNS displacement assays were performed with AtDIR1, AtDIR1-like, CsDIR1, CsDIR2, and AtLTP2.12 proteins and commercially available SAR signal molecules, AzA, pipecolic acid (Pip), and G3P, as well as a buffer control (MES). Fluorescence was measured before and after the addition of each SAR molecule to a mixture of recombinant protein and TNS. If a putative ligand enters the LTP hydrophobic cavity, TNS molecules are displaced and fluorescence decreases. No significant differences in TNS fluorescence were observed for AtLTP2.12, AtDIR1 and AtDIR1-like, regardless of whether MES or SAR signal molecules were added (**Figure 6**). Interestingly, the addition of AzA lead to reduced TNS fluorescence in both CsDIR1 (∼25% lower than the MES control) and CsDIR2 (∼30% lower), while the addition of Pip to CsDIR1 resulted in a similar reduction in TNS fluorescence (∼25% lower). This suggests that features of the CsDIR1 and CsDIR2 hydrophobic cavities allow for modest binding of AzA or Pip *in vitro*. Alternatively, Pip and AzA may interact outside of the cavity, resulting in a conformational change that modestly impacts the TNS binding of CsDIR1/2. In contrast, interaction of AtDIR1 with these SAR signals was not observed, perhaps because AtDIR1 requires *in vivo* factors to facilitate signal binding.

### Validation of *In Silico* Orthology Analysis Using the Cucumber and *Arabidopsis* Model Systems

**FIGURE 7:**
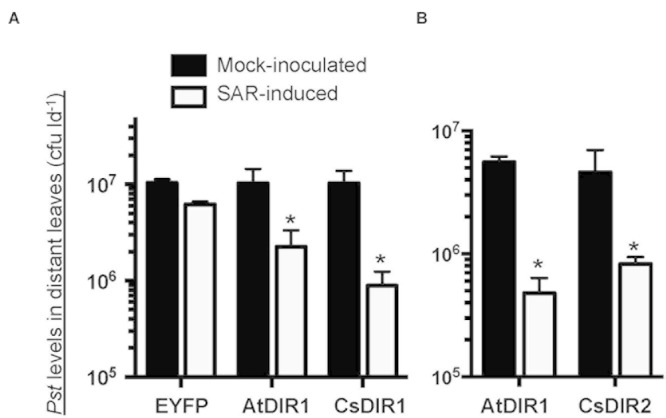
***In vivo* complementation of *dir1-1* by *Agrobacterium*-mediated transient expression of **(A)***CsDIR1* and **(B)***CsDIR2*.** At 3 weeks post germination (wpg), two leaves of *dir1-1* were inoculated with *Agrobacterium* expressing EYFP, AtDIR1(-EYFP), CsDIR1, or CsDIR2. Four days later, the same leaves were mock-inoculated (10 mM MgCl2) or induced for SAR with 10^6^ cfu ml^-1^ *Pst* (*avrRpt2*). Distant leaves were challenged for SAR 2 days post mock/SAR-induction with virulent *Pst* (10^5^ cfu ml^-1^) and *in planta* bacterial levels were determined in distant leaves 3 days post challenge. Statistically significant differences between mock-induced and SAR-induced plants (Student’s *t*-test *p* < 0.05) are indicated with asterisks (^∗^). **(A)** was performed four times with similar results and **(B)** was performed three times with similar results.

To validate our *in silico* orthology data (**Figures 1** and **2**; **Table 1**) we investigated the role of DIR1 during SAR in cucumber by combining the *Arabidopsis* and cucumber SAR model systems (Rasmussen et al., 1991; Cameron et al., 1994, 1999) in two ways. First, we reasoned that if cucumber and *Arabidopsis* DIR1 proteins are functionally equivalent, transiently expressed CsDIR1/2 proteins should rescue the SAR-defective *dir1-1 Arabidopsis* mutant. The *Agrobacterium*-mediated transient-SAR (Agro-SAR) assay previously developed in our lab (Champigny et al., 2013) was used to transiently express *CsDIR1* or *CsDIR2* in one leaf of *dir1-1*. *Agrobacterium*-mediated transient expression of AtDIR1 and EYFP was included as a positive and negative control, respectively. RT-PCR analysis confirmed successful Agro-mediated transient expression of each transgene in *dir1-1* leaves 4 days post agro-infiltration (**Supplementary Figure S3**). Four days after *Agrobacterium* inoculation, leaves were induced for SAR using 10^6^ cfu ml^-1^ *Pst(avrRpt2)*. The SAR response was measured in distant leaves at 3 dpi with 10^5^ cfu ml^-1^ virulent *Pst*. The positive control gave the expected result in that transient expression of AtDIR1 complemented the *dir1-1* mutation, as indicated by a significant ∼fivefold reduction (Student’s *t*-test, *p* < 0.05) in bacterial density in distant leaves of SAR-induced plants compared to mock-inoculated control plants (**Figure 7A**). Expression of EYFP did not complement the SAR defect in *dir1-1*, confirming that *Agrobacterium* infection did not induce the SAR response (**Figure 7A**). Expression of *CsDIR1* in one leaf of the *dir1-1* mutant resulted in a significant ∼12-fold reduction in *Pst* levels in SAR-induced versus mock-inoculated plants (**Figure 7A**), while expression of *CsDIR2* in a separate experiment resulted in a significant ∼sixfold reduction (**Figure 7B**). We concluded that transient expression of CsDIR1 and CsDIR2 complemented the *dir1-1* SAR defect, providing evidence that both cucumber orthologs are functionally similar to AtDIR1.

**FIGURE 8:**
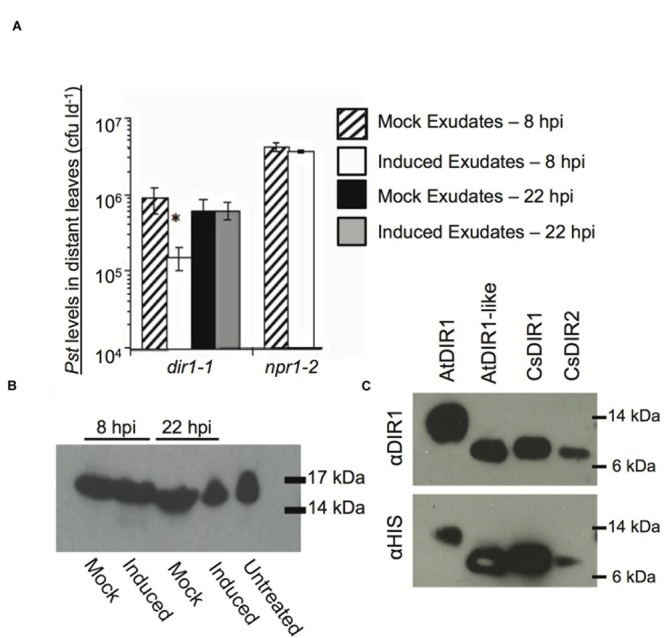
**Characterization of DIR1-mediated SAR in *Cucumis sativus*. (A)** SAR-induced cucumber exudates rescue the SAR defect in *dir1-1.* Petiole exudates were collected from cut cucumber ends at 8 or 22 hpi with 10 mM MgCl_2_ (mock) or SAR-inducing (induced) *Pseudomonas syringae* pv. *syringae* D20. Exudates from Mock (8 or 22 hpi) or SAR-induced (8 or 22 hpi) were infiltrated into 2 *dir1-1* or *npr1-2* leaves. Two days later distant leaves were inoculated with virulent *Pst* (10^5^ cfu ml*-1*) and 3 dpi *Pst* levels were measured. An asterisk (^∗^) indicates a significant difference between plants infiltrated with mock and induced exudates. **(B)** A DIR1-sized band is present in cucumber phloem before and after SAR induction Exudates were lyophilized and subjected to immunoblot analysis using the DIR1 antibody. 17 and 14 kDa protein molecular weight markers are indicated. **(C)** Recombinant CsDIR1 and CsDIR2 protein is detected by the polyclonal DIR1 antibody. Immunoblots of crude extracts from IPTG-induced Rosetta-gami strains expressing His-tagged AtDIR1, AtDIR1-like, CsDIR1, and CsDIR2 proteins using anti-DIR1 or anti-HIS antibodies. **(A)** Was repeated two additional times, **(B,C)** were repeated once, all with similar results.

We further combined the cucumber and *Arabidopsis* SAR models to examine the importance of CsDIR1/2 during SAR and to determine if cucumber DIR1 proteins act as SAR long distance signals in cucumber. Phloem exudates collected from mock- and SAR-induced cucumber leaves were collected and infiltrated into the *dir1-1* and *npr1-2 Arabidopsis* SAR mutants to determine whether SAR-induced cucumber exudates contain SAR signals capable of rescuing these mutants. In cucumber, SAR signals move out of induced leaves between 4 and 8 hpi (Rasmussen et al., 1991). Therefore, cucumber phloem exudates were collected at two time-points after inoculation with 10^8^ cfu ml^-1^ *P. syringae* pv *syringae* D20 (*Pss*): at 8 hpi when SAR signals are accumulating and at 22 hpi when SAR signals are no longer present in the phloem. Cucumber exudates containing 5–15 mg ml^-1^ protein were diluted 15-fold and infiltrated into two lower leaves of *dir1-1* or *npr1-2* (negative control). Two days later, distant *Arabidopsis* leaves were inoculated with 10^5^ cfu ml^-1^ virulent *Pst DC3000* and bacterial levels were measured 3 dpi to assay for SAR competence. The *npr1-2* mutant is defective in acting on mobile SAR signals in systemic leaves (Cao et al., 1997; Fu and Dong, 2013). Therefore, as expected, SAR was not established in *npr1-2* distant leaves after infiltration of SAR-induced (8 hpi) cucumber exudates, as demonstrated by similar *Pst* levels in plants that received exudates collected from either mock-inoculated or SAR-induced leaves (**Figure 8A**). SAR was established in *dir1-1* distant leaves after infiltration of SAR-induced cucumber exudates (8 hpi), as demonstrated by a sixfold reduction in *Pst* levels compared to plants that were infiltrated with exudates collected from mock-inoculated leaves or inactive (22 hpi) exudates (**Figure 8A**). Similar to previous reports (Rasmussen et al., 1991; Smith-Becker et al., 1998), SA was not detected in the 8 hpi cucumber exudates (data not shown), ruling out the possibility that SA present in the exudates induced the observed resistance. These experiments suggest that cucumber petiole exudates collected at 8 hpi, but not at 22 hpi contain SAR long-distance signals that rescue the *dir1-1* SAR defect, and that mobile SAR signals are conserved between cucumber and *Arabidopsis*. Moreover, this provides additional evidence that the cucumber genome contains DIR1 orthologs that can compensate for the absence of AtDIR1 protein in the *dir1-1* mutant.

Rescue of the *dir1-1* SAR defect by transient expression of CsDIR1/2 or by infiltration of SAR-induced cucumber exudates suggested that cucumber DIR1 orthologs function like AtDIR1 during SAR. To obtain additional support for this idea, we investigated whether the AtDIR1 antibody detected orthologous CsDIR1/2 proteins in cucumber petiole exudates. Petiole exudates collected from mock- (10 mM MgCl_2_) and SAR-induced (*Pss* D20) cucumber leaves at 8 hpi and 22 hpi were subjected to immunoblot analysis with the AtDIR1 antibody. A ∼15 kDa band was detected in untreated, mock-inoculated and SAR-induced petiole exudates, suggesting that a protein related to AtDIR1 is constitutively present in cucumber phloem (**Figure 8B**). To confirm that the AtDIR1 antibody detects CsDIR1 and/or CsDIR2, these proteins, along with AtDIR1 and AtDIR1like controls, were ectopically expressed in Rosetta Gami *E. coli*. Immunoblots revealed that, as with AtDIR1 and AtDIR1like, the AtDIR1 antibody detected both CsDIR1 and CsDIR2 proteins (**Figure 8C**). This suggests that the proteins detected by the AtDIR1 antibody in the cucumber phloem samples (**Figure 8B**) represent CsDIR1 and/or CsDIR2, providing further support for the hypothesis that CsDIR1/2 act as long-distance signals during SAR.

## Discussion

### Orthology Analysis Identifies Conserved Residues in DIR1

DIR1 is hypothesized to bind and translocate a lipidic signal during SAR. In support of this hypothesis, *in vitro* experiments have demonstrated the non-specific loading of fatty acids into the hydrophobic pocket of DIR1 (Lascombe et al., 2008). However, *in vivo* evidence supporting this or any biochemical function during SAR has not been reported. In this study, we identified and compared DIR1 orthologs to reveal conserved residues or motifs. Many conserved residues among DIR1 orthologs were involved in the formation of the central hydrophobic cavity. This result is not surprising, as LTPs are small proteins whose defining feature is a central cavity that can accommodate fatty acids. We generated DIR1 variant proteins to determine the structural importance of each conserved residue/motif. This was achieved using an *in vitro* binding assay based on the fluorescent lipophilic probe TNS. As described previously, the TNS probe fluoresces only in hydrophobic environments (McClure and Edelman, 1966). Since LTPs are small proteins with a central hydrophobic cavity, TNS fluorescence in the presence of an LTP is attributed to loading of TNS into the cavity. As such, this assay is often used as an indicator of LTP folding status and lipid-binding capacity *in vitro* (Mikes et al., 1998; Girault et al., 2008; DeBono et al., 2009; Choi et al., 2012; Guo et al., 2013). Among the DIR1 variants tested, a significant reduction in TNS-binding was observed in the D39Q, L43D, and DIR1^ΔCys^ proteins compared to the wild-type AtDIR1 protein. The largest reduction in TNS-binding was observed in the DIR1^ΔCys^ protein, which lacks the eight cysteine residues responsible for the generation of four disulphide bonds that establish the hydrophobic cavity. This variant displayed reduced TNS-fluorescence levels that were comparable to those observed with denatured AtDIR1, indicating that the DIR1^ΔCys^ protein does not contain the hydrophobic cavity. Both the L43D and D39Q variants also displayed reduced TNS-binding compared to the wild-type AtDIR1. Leucine 43 is located between two cysteine residues, a position that is hypothesized to be important for cysteine bond pairing (Samuel et al., 2002). In the L43D DIR1 variant, the conserved non-polar leucine residue was modified to a polar aspartic acid residue. This modification resulted in reduced TNS-fluorescence compared to that observed with AtDIR1, suggesting that this residue is important for hydrophobic ligand interactions, or that it contributes to the size or shape of the hydrophobic cavity. A similar effect was reported in mutagenesis studies of the rice (*Oryza sativa*) OsLTP2 protein, where modifying the same position from phenylalanine to alanine (F36A) disrupted the formation of the hydrophobic cavity, as determined by circular dichroism, NMR spectroscopy, and a fluorescence-based assay using the ANS (1-anilino-8-naphthalene sulfonate) probe (Cheng et al., 2008). Using the comparable TNS binding assay, we observed similar binding defects for the L43D AtDIR1 variant as was observed for the OsLTP2 F36A variant (Cheng et al., 2008). The conserved D39 (aspartic acid) residue was modified to determine the importance of the DIR1-specific AD motif present at the bottom of the DIR1 cavity. In this variant, the charged aspartic acid residue was modified to a similarly shaped but non-charged glutamine residue. TNS binding was affected to a small degree in the D39Q variant compared to the wild-type protein, suggesting that this residue contributes to ligand binding. Alternatively, the AD motif could be the site of an adduct formation with potential SAR signals/ligands, as a similarly exposed aspartic acid residue in the barley LTP1 protein is the site of allene oxide adduct formation (Bakan et al., 2006).

The other DIR1 variants (AxxAxxA, F40Y, and NPH) displayed similar TNS-binding profiles to that of wild-type AtDIR1. The NPH variant lacks three polar residues (Gln9, Asn13, Lys16) at the entrance of the hydrophobic cavity. It is not surprising that that TNS-binding was unaffected in this variant as these exterior residues are thought to stabilize a ligand possessing a hydrophilic moiety that remains outside of the hydrophobic cavity. In contrast, the F40 phenylalanine residue is located within the central hydrophobic cavity. AtDIR1-like, which is hypothesized to have a reduced capacity to participate in SAR (Champigny et al., 2013), contains a polar tyrosine residue at position 40, while AtDIR1 and other orthologs contain a non-polar phenylalanine residue. In this study, we demonstrated that the F40Y and AtDIR1-like proteins have similar TNS-binding curves to that of AtDIR1, indicating that the F40 residue does not affect the ability of the cavity to bind TNS molecules. As outlined previously (Champigny et al., 2013), we hypothesize that the F40Y substitution decreases the affinity of DIR1-like for a specific ligand, thereby compromising its ability to participate in SAR.

The conserved PxxPxxP and LAxxLP motifs are located on the exterior of AtDIR1 and are thought to contribute little to cavity shape, size or ligand interaction. The PxxPxxP motif is predicted to mediate protein–protein interactions (Lascombe et al., 2008), however PxxP motifs are thought to interact with SH3 domain-containing proteins (Li, 2005). It is possible that DIR1 interacts with one of the three predicted SH3 domain-containing proteins (AtSH3P1-3) in *Arabidopsis* (Lam et al., 2001), or that the mechanism of PxxP-based protein–protein interaction in *Arabidopsis* is distinct from the classical PxxP–SH3 interaction. Lastly, the LAxxLP motif is located on the surface of DIR1 and may also be involved in mediating, or maintaining, protein–protein interactions.

### DIR1 Is Conserved in Tobacco, Tomato, Cucumber, and Soybean

DIR1 is known to play a significant role during SAR in *Arabidopsis*; however, its importance in other plants has not been well defined. Mitton et al. (2009) identified a putative DIR1 ortholog in tomato, SlDIR1 (SGN-U584000, formerly LeDIR1; SGN-327306). SlDIR1 is 63% similar to mature AtDIR1 at the amino acid level and has a similarly low pI of 4.03. Interestingly, SlDIR1 is present in petiole exudates of untreated tomato leaves (Mitton et al., 2009), whereas AtDIR1 only accumulates to detectable levels in petiole exudates collected from local and distant leaves of SAR-induced plants (Champigny et al., 2013; Shah et al., 2014; Carella et al., 2015). Nevertheless, the presence of SlDIR1 in petiole exudates suggests that it participates in long-distance SAR signaling, and thus may function similarly to AtDIR1. A previous report hinted at the functional redundancy of SAR signals between *Arabidopsis* and tomato, as infiltration of petiole exudates collected from SAR-induced leaves of *Arabidopsis* induced SAR in tomato (Chaturvedi et al., 2008). We hypothesize that SlDIR1 (and/or SlDIR2/3) participates in tomato long-distance SAR signaling.

A more comprehensive study demonstrated that DIR1-mediated SAR signaling is conserved in tobacco. Of the three *N. tabacum* DIR1 orthologs, NtDIR2 and NtDIR3 complemented the SAR-defect in *Arabidopsis dir1-1*, demonstrating that these two orthologs (which have 57 and 67% protein sequence similarity, respectively, to AtDIR1) have a similar function to AtDIR1 (Liu et al., 2011). Knockdown of *NtDIR2/3* expression in tobacco RNAi lines resulted in a loss of SAR to TMV (tobacco mosaic virus), which was associated with heightened *SAMT1* (Salicylic Acid Methyl-transferase) expression and increased MeSA levels compared to wild-type plants. This was consistent with the observation that *dir1-1* mutants have increased MeSA levels and higher *BSMT1* (Benzoic acid/Salicylic acid Carboxyl Methyl-transferase) expression after pathogen infection relative to wild-type plants (Liu et al., 2011). Interestingly, NtDIR1, which shares 62% amino acid similarity with AtDIR1, was unable to complement *Arabidopsis dir1-1* and NtDIR1-RNAi tobacco lines with reduced NtDIR1 expression (but wild-type NtDIR2/3 expression levels), were SAR-competent. Although NtDIR2/3 movement into petiole exudates during SAR was not determined, the authors suggested that NtDIR2/3 are translocated through the phloem to activate SAR in distant tobacco leaves (Liu et al., 2011). Taken together, the existence of DIR1 orthologs in several crop plants, and the conservation of DIR1 function in tobacco and cucumber, suggests that DIR1-mediated SAR is important in a number of plant species.

### DIR1-Mediated SAR in *Cucumis sativus*

Two putative DIR1 orthologs are encoded in the *C. sativus* genome, CsDIR1 and CsDIR2, which share 61 and 65% amino acid sequence similarity, respectively, with mature AtDIR1. Homology models of the CsDIR1/2 proteins identified structural similarities with AtDIR1. TNS-binding experiments demonstrated that CsDIR1, CsDIR2, and AtDIR1 have statistically similar affinities for the lipophilic TNS probe, confirming that these proteins are indeed structurally similar. Phloem exudate rescue and Agro-SAR rescue experiments demonstrated that DIR1-mediated SAR is conserved in cucumber. Interestingly, proteins that cross-reacted with the AtDIR1-antibody were detected in phloem exudates collected from both mock- and SAR-induced cucumber leaves at 8 and 22 hpi. Accumulation of the LeDIR1 protein in petiole exudates collected from untreated tomato plants was also observed (Mitton et al., 2009). However, DIR1-antibody signals were only detected in *Arabidopsis* exudates after SAR induction (Champigny et al., 2013; Shah et al., 2014; Carella et al., 2015). Importantly, only exudates from SAR-induced cucumber leaves collected at 8 hpi, when SAR signals are present, were able to induce SAR in the *Arabidopsis dir1-1* mutant, even though DIR1-antibody cross-reacting proteins were present in all exudates. This result supports the idea that both cucumber and *Arabidopsis* DIR1 are activated during SAR-induction, perhaps by binding a ligand(s) and/or becoming part of a mobile signal complex.

### Searching for DIR1 Ligands

We used an *in vitro* TNS-based fluorescence displacement assay to determine whether AtDIR1 can interact with known inducers of SAR (G3P, AzA, Pip). Although these molecules do not resemble typical fatty acid ligands of LTPs *in vitro*, we examined their ability to displace TNS from the hydrophobic cavity of DIR1 because these signals are present in the phloem during SAR induction, along with DIR1. Moreover, G3P and AzA both require DIR1 for their resistance-inducing activity (Jung et al., 2009; Chanda et al., 2011). AtDIR1 and AtDIR1like failed to interact with any of the SAR inducers, suggesting that these molecules are not DIR1 ligands. However, it also possible that SAR induction causes DIR1 or AzA/G3P/Pip modification and/or the formation of a SAR signal complex *in planta* that is required for DIR1–ligand interaction. Alternatively, DIR1 may bind a different SAR-inducing molecule or an unknown ligand. Given that DIR1-containing high molecular weight complexes co-fractionate with the diterpenoid SAR-inducer dehydroabietinal in phloem exudates collected from SAR-induced *Arabidopsis* leaves (Chaturvedi et al., 2012; Shah et al., 2014), we speculate that DIR1 may interact with dehydroabietinal. Moreover, DIR1’s association with a high molecular weight complex may indicate that other proteins are required for DIR1-ligand binding, which could explain why purified recombinant AtDIR1 did not interact with the tested SAR signaling molecules.

Intriguingly, the TNS displacement assays suggest that AzA and Pip displace TNS from the hydrophobic cavity of CsDIR1, while AzA displaces TNS from the CsDIR2 cavity. This leads us to speculate that AzA and Pip may contribute to DIR1 function in cucumber, as ligands that enter the DIR1 hydrophobic cavity. Alternatively, AzA and Pip may act outside of the hydrophobic cavity to cause allosteric effects that alter the shape or size of the cavity. It is currently unknown whether AzA or Pip accumulate in phloem exudates during SAR induction in cucumber, therefore further experimentation is needed to characterize the role of these SAR signal molecules during the cucumber SAR response.

## Conclusion

DIR1 orthology analysis identified amino acid motifs (L43, AD, and the eight cysteine motif) that are important for TNS binding, supporting the hypothesis that they contribute to the size, shape, or lipid binding ability of the hydrophobic cavity, an essential feature for hydrophobic ligand–LTP interaction. In addition, we developed an *Arabidopsis*–cucumber SAR model to further explore the role of DIR1 during SAR. Using this model, we discovered two cucumber orthologs that function similarly to AtDIR1 during SAR. Although DIR1-antibody signals are constitutively present in cucumber phloem sap, only SAR-induced cucumber phloem exudates rescued the SAR defect in *dir1-1.* Together, these data suggest that DIR1-mediated SAR signaling is conserved in cucumber, further demonstrating the importance of DIR1 in long-distance systemic immune signaling in plants.

## Author Contributions

MI and PC contributed equally as first authors. Designed experiments: MI, PC, MC, JR, and RC. Performed experiments: MI, PC, MC, and JF. Analyzed data: MI, PC, MC, JF, and RC. Provided reagents and equipment: JR and RC. PC and RC wrote the bulk of the manuscript, with significant contributions by MI and JR.

## Conflict of Interest Statement

The authors declare that the research was conducted in the absence of any commercial or financial relationships that could be construed as a potential conflict of interest.

## Footnotes

1 http://www.arabidopsis.org

2 http://www.ncbi.nlm.nih.gov/

3 http://phytozome.net

4 http://www.ebi.ac.uk/emboss/align

5 http://www.cbs.dtu.dk/services/SignalP

6 http://www.expasy.org/spdbv/

7 http://weblogo.berkeley.edu

## Supplementary Material

FIGURE S1 **Individual *in vitro* TNS (6,*P*-toluidinylnaphthalene-2-sulfonate) binding assays of recombinant AtDIR1, AtDIR1-like, AtLTP2.12, CsDIR1, CsDIR2, and DIR1 variant proteins.** Natured protein (black lines, circles) is compared to denatured protein for sample (gray lines, squares). Increasing concentrations of TNS were added to each Rosetta Gami *E. coli* purified protein lacking the ER signal sequence. TNS binding curves were generated in PRISM6 by non-linear curve fitting for one site saturation binding. Proteins were denatured by boiling in 6 M Urea. Samples were excited at 320 nm and emission at 437 nm and the change in fluorescence was calculated for three replicates. Error bars represent the standard deviation. An asterisk (^∗^) indicates proteins that were analyzed using a TECAN M1000 rather than the Gen5 BioTek fluorometer.

FIGURE S2 **Batch Reproducibility of the TNS binding assay.** TNS binding curves of identical proteins purified from different batches was performed for AtDIR1 **(A)** and AtDIR1- like **(B)**. Student’s *t*-tests (*p* < 0.05) of natured proteins from batch 1 and batch 2 did not identify statistically significant differences in TNS binding capacity (means of denatured proteins were not compared). Values represent the mean ± standard deviation of 3 technical replicates. These assays were performed using a Gen5 Synergy 4 (BioTek) plate reader.

FIGURE S3 **Confirmation of successful *Agrobacterium*-mediated expression in *dir1-1*.** RT-PCR analysis was used to monitor the expression of *EYFP, AtDIR1-EYFP, CsDIR1*, and *CsDIR2* four days post agro-infiltration of *dir1-1* leaves (3.5 week-old plants). Expression of the endogenous *ACTIN1* housekeeping gene was monitored as a loading control. Primers for RT-PCR analysis can be found in Table S1. This was performed twice with similar results.

## References

[B1] Arnold K. Bordoli L. Kopp J. Schwede T. (2006). The SWISS-MODEL workspace: a web-based environment for protein structure homology modelling. *Bioinformatics* 22 195–201. 10.1093/bioinformatics/bti770 16301204

[B2] Bakan B. Hamberg M. Perrocheau L. Maume D. Rogniaux H. Tranquet O. (2006). Specific adduction of plant lipid transfer protein by an allene oxide generated by 9-lipoxygenase and allene oxide synthase. *J. Biol. Chem.* 281 38981–38988. 10.1074/jbc.M608580200 17046828

[B3] Buhot N. Gomes E. Milat M.-L. Ponchet M. Marion D. Lequeu J. (2004). Modulation of the biological activity of a tobacco LTP1 by lipid complexation. *Mol. Biol. Cell* 15 5047–5052. 10.1091/mbc.E04-07-0575 15356262 PMC524770

[B4] Cameron R. K. Dixon R. A. Lamb C. J. (1994). Biologically induced systemic acquired resistance in *Arabidopsis thaliana*. *Plant J.* 5 715–725. 10.1111/j.1365-313X.1994.00715.x

[B5] Cameron R. K. Paiva N. L. Lamb C. J. Dixon R. A. (1999). Accumulation of salicylic acid and PR-1 gene transcripts in relation to the systemic acquired resistance (SAR) response induced by *Pseudomonas* syringae pv. tomato in *Arabidopsis*. *Phys. Mol. Plant Pathol.* 55 121–130. 10.1006/pmpp.1999.0214

[B6] Cao H. Glazebrook J. Clark J. D. Volko S. Dong X. (1997). The *Arabidopsis* NPR1 gene that controls systemic acquired resistance encodes a novel protein containing ankyrin repeats. *Cell* 88 57–63. 10.1016/S0092-8674(00)81858-9 9019406

[B7] Carella P. Isaacs M. Cameron R. K. (2015). Plasmodesmata-located protein overexpression negatively impacts the manifestation of systemic acquired resistance and the long-distance movement of defective in induced resistance 1 in *Arabidopsis*. *Plant Biol.* 17 395–401. 10.1111/plb.12234 25296648

[B8] Cecchini N. M. Steffes K. Schlappi M. R. Gifford A. N. Greenberg J. T. (2015). Arabidopsis AZI1 family proteins mediate signal mobilization for systemic defence priming. *Nat. Commun.* 6:7658 10.1038/ncomms8658 26203923

[B9] Champigny M. J. Cameron R. K. (2009). “Action at a distance: long- distance signals in induced resistance,” in *Plant Innate Immunity* Vol. 51 ed. Van Loon L. C. (London: Academic Press) 123–171.

[B10] Champigny M. J. Isaacs M. Carella P. Faubert J. Fobert P. Cameron R. K. (2013). Long distance movement of DIR1 and investigation of the role of DIR1-like during systemic acquired resistance in *Arabidopsis*. *Front. Plant Sci.* 4:230 10.3389/fpls.2013.00230 PMC3701462 23847635

[B11] Champigny M. J. Shearer H. Mohammed A. Haines K. Neumann M. Thilmony R. (2011). Localization of DIR1 at the tissue, cellular, and subcellular levels during systemic acquired resistance in *Arabidopsis* using DIR1: GUS and DIR1:EGFP reporters. *BMC Plant Biol.* 11:125 10.1186/1471-2229-11-125 PMC3180652 21896186

[B12] Chanda B. Xia Y. Mandal M. K. Yu K. Sekine K.-T. Gao Q.-M. (2011). Glycerol-3-phosphate is a critical mobile inducer of systemic immunity in plants. *Nat. Genet.* 43 421–427. 10.1038/ng.798 21441932

[B13] Chaturvedi R. Krothapalli K. Makandar R. Nandi A. Sparks A. A. Roth M. R. (2008). Plastid w3-fatty acid desaturase-dependent accumulation of a systemic acquired resistance inducing activity in petiole exudates of *Arabidopsis thaliana* is independent of jasmonic acid. *Plant J.* 71 161–172. 10.1111/j.1365-313X.2007.03400.x 18088304

[B14] Chaturvedi R. Venables B. Petros R. A. Nalam V. Li M. Wang X. (2012). An abietane diterpenoid is a potent activator of systemic acquired resistance. *Plant J.* 71 161–172. 10.1111/j.1365-313X.2012.04981.x 22385469

[B15] Cheng C. S. Chen M. N. Lai Y. T. Chen T. Lin K. F. Liu Y. J. (2008). Mutagenesis study of rice non-specific lipid transfer protein 2 revels residues that contribute to structure and ligand binding. *Proteins* 70 695–706. 10.1002/prot.21520 17729272

[B16] Choi Y. E. Lim S. Kim H.-J. Han J. Y. Lee M.-H. Yang Y. (2012). Tobacco NtLTP1, a glandular-specific lipid transfer protein, is required for lipid secretion from glandular trichomes. *Plant J.* 70 480–491. 10.1111/j.1365-313X.2011.04886.x 22171964

[B17] Crooks G. E. Hon G. Chandonia J. Brenner S. E. (2004). Weblogo: a sequence logo generator. *Genome Res.* 14 1188–1190. 10.1101/gr.849004 15173120 PMC419797

[B18] Curtis M. D. Grossniklaus U. (2003). A gateway cloning vector set for high-throughput functional analysis of genes in planta. *Plant Physiol.* 133 462–469. 10.1104/pp.103.027979 14555774 PMC523872

[B19] DeBono A. Yeats T. H. Rose J. K. C. Bird D. Jetter R. Kunst L. (2009). *Arabidopsis* LTPG is a glycosylphosphatidylinositol-anchored lipid transfer protein required for export of lipids to the plant surface. *Plant Cell* 21 1230–1238. 10.1105/tpc.108.064451 19366900 PMC2685631

[B20] Dempsey D. M. Klessig D. F. (2012). SOS – too many signals for systemic acquired resistance? *Trends Plant Sci.* 17 538–545. 10.1016/j.tplants.2012.05.011 22749315

[B21] Desormeaux A. Blochet J.-E. Pezolet M. Marion D. (1992). Amino acid sequence of a non-specific what phospholipid transfer protein and its conformation as revealed by infrared and raman spectroscopy. Role of disulfide bridges and phospholipids in the stabilization of the alpha-helix structure. *Biochim. Biophys*. *Acta* 1121 137–152. 10.1016/0167-4838(92)90347-G 1599935

[B22] Douliez J. P. Michon T. Elmorjani K. Marion D. (2000). Structure, biological and technological functions of lipid transfer proteins and indolines, the major lipid binding proteins from cereal kernals. *J. Cereal Sci.* 32 1–20. 10.1006/jcrs.2000.0315

[B23] Drummond A. J. Suchard M. A. Dong X. Rambaut A. (2012). Bayesian phylogentics with BEAUti and the BEAST 1.7. *Mol. Biol. Evol.* 29 1969–1973. 10.1093/molbev/mss075 22367748 PMC3408070

[B24] Edstam M. M. Viitanen L. Salminen T. A. Edqvist J. (2011). Evolutionary history of the non-specific lipid transfer proteins. *Mol. Plant* 4 947–964. 10.1093/mp/ssr019 21486996

[B25] Felsenstein J. (1985). Confidence limits on phylogenies: an approach using the bootstrap. *Evolution* 39 783–791. 10.2307/2408678 28561359

[B26] Fu Z. Q. Dong X. (2013). Systemic acquired resistance: turning local infection into global defense. *Annu. Rev. Plant Biol.* 64 839–863. 10.1146/annurev-arplant-042811-105606 23373699

[B27] Girault T. Fancais J. Rogniaux H. Pascal S. Delrot S. Coutos-Thevenot P. (2008). Exogenous application of a lipid transfer protein-jasmonic acid complex induces protection against grapevine towards infection by *Botrytis cinerea*. *Plant Physiol. Biochem.* 46 140–149. 10.1016/j.plaphy.2007.10.005 18023196

[B28] Goodstein D. M. Shu S. Howson R. Neupane R. Hayes R. D. Fazo J. (2012). Phytozome: a comparative platform for green plant genomics. *Nucleic Acid Res.* 40 D1178–D1186. 10.1093/nar/gkr944 22110026 PMC3245001

[B29] Guedes M. E. M. Richmond S. Kuc J. (1980). Induced systemic resistance to anthracnose in cucumber as influenced by the location of the inducer inoculation with *Colletotrichum lagenarium* and the onset of flowering and fruiting. *Physiol. Plant Pathol.* 17 229–233. 10.1016/0048-4059(80)90056-9

[B30] Guex N. Peitsch M. C. (1997). SWISS-MODEL and the Swiss-Pdb viewer: an environment for comparative protein modeling. *Electrophoresis* 18 2714–2723. 10.1002/elps.1150181505 9504803

[B31] Guo L. Yang H. Zhang X. Yang S. (2013). Lipid transfer protein 3 as a target of MYB96 mediates freezing and drought stress in *Arabidopsis*. *J. Exp. Bot.* 64 1755–1767. 10.1093/jxb/ert040 23404903 PMC3617838

[B32] Han M. V. Zmasek C. M. (2009). phyloXML: XML for evolutionary biology and comparative genomics. *BMC Bioinformat.* 10:356 10.1186/1471-2105-10-356 PMC2774328 19860910

[B33] Hoh F. Pons J. Fautier M. Lamotte F. D. Dumas C. (2005). Structure of a liganded type 2 non-specific lipid-transfer protein from wheat and the molecular basis of lipid binding. *Acta Crystallogr. D Biol. Crystallogr.* 61 397–406. 10.1107/S0907444905000417 15805594

[B34] Jung H. W. Tschaplinski T. J. Wang L. Glazebrook J. Greenberg J. T. (2009). Priming in systemic immunity. *Science* 324 89–91. 10.1126/science.1170025 19342588

[B35] Kiefer F. Arnold K. Kunzli M. Mordoli L. Schwede T. (2009). The SWISS-MODEL repository and associated resources. *Nucleic Acids Res.* 37 387–392. 10.1093/nar/gkn750 PMC2686475 18931379

[B36] Kiefer I. W. Slusarenko A. J. (2003). The pattern of systemic acquired resistance induction within the *Arabidopsis* rosette in relation to the pattern of translocation. *Plant Physiol.* 132 840–847. 10.1104/pp.103.021709 12805614 PMC167024

[B37] Kimura M. (1980). A simple method for estimating evolutionary rate of base substitutions through comparative studies of nucleotide sequences. *J. Mol. Evol.* 16 111–120. 10.1007/BF01731581 7463489

[B38] King E. O. Ward M. K. Raney D. E. (1954). Two simple media for the demonstration of pyocyanin and fluorescin. *J. Lab. Clin. Med.* 44 301–307. 13184240

[B39] Krasikov V. Dekker H. Rep M. Takken F. L. W. (2011). The tomato xylem sap protein XSP10 is required for full susceptibility to fusarium wilt disease. *J. Exp. Bot.* 62 963–973. 10.1093/jxb/erq327 20974736 PMC3022394

[B40] Lam B. C. H. Sage T. L. Bianchi F. Blumwald E. (2001). Role of SH3 domain-containing proteins in clathrin-mediated vesicle trafficking in *Arabidopsis*. *Plant Cell* 13 2499–2512. 10.1105/tpc.13.11.2499 11701884 PMC139467

[B41] Lascombe M. Bakan B. Buot N. Marion D. Blein J. Larue V. (2008). The structure of “defective in induced resistance” protein of *Arabidopsis thaliana*, DIR1, reveals a new type of lipid transfer protein. *Protein Sci.* 17 1522–1530. 10.1110/ps.035972.108 18552128 PMC2525531

[B42] Lascombe M. Buhot N. Bakan B. Marion D. Blein J. P. Lamb C. J. (2006). Cystallization of DIR1, a LTP2-like resistance signaling protein from *Arabidopsis thaliana*. *Acta Crystallogr. Sect F Struct. Biol. Cryst. Commun.* 62 702–704. 10.1107/S1744309106023748 PMC2242958 16820699

[B43] Lei L. Chen L. Shi X. Li Y. Wang J. Chen D. (2014). A nodule-specific lipid transfer protein AsE246 participates in transport of plant-synthesized lipids to symbiosome membrane and is essential for nodule organogenesis in chinese milk vetch. *Plant Physiol.* 164 1045–1058. 10.1104/pp.113.232637 24367021 PMC3912078

[B44] Li B.-C. Zhang C. Chai Q.-X. Han Y.-Y. Wang X.-Y. Liu M.-X. (2014). Plasmalemma localisation of double hybrid proline-rich protein 1 and its function in systemic acquired resistance of *Arabidopsis thaliana*. *Funct. Plant Biol.* 41 768–779. 10.1071/FP13314 32481031

[B45] Li S. S.-C. (2005). Specificity and versatility of SH3 and other proline-rich recognition domains: structural basis and implications for cellular signal transduction. *Biochem. J.* 390 641–653. 10.1042/BJ20050411 16134966 PMC1199657

[B46] Liu P.-P. von Dahl C. C. Park S.-W. Klessig D. F. (2011). Interconnection between methyl salicylate and lipid-based long-distance signaling during the development of systemic acquired resistance in *Arabidopsis* and tobacco. *Plant Physiol.* 155 1762–1768. 10.1104/pp.110.171694 21311035 PMC3091099

[B47] Maldonado A. M. Doerner P. Dixon R. A. Lamb C. J. Cameron R. K. (2002). A putative lipid transfer protein involved in systemic resistance signalling in *Arabidopsis*. *Nature* 419 399–403. 10.1038/nature00962 12353036

[B48] McClure W. O. Edelman G. M. (1966). Fluorescent probes for conformational states of proteins, I. *Mechanism of fluorescence* of 2-p-toluidinylaphthalene-6sulfonate, a hydrophobic probe. *Biochemistry* 5 1908–1919. 10.1021/bi00870a018 4164420

[B49] Mikes V. Milat M.-L. Ponchet M. Panabieres F. Ricci P. Blein J.-P. (1998). Elicitins, proteinaceous elicitors of plant defense, are a new class of sterol carrier proteins. *Biochem. Biophys. Res. Commun.* 245 133–139. 10.1006/bbrc.1998.8341 9535796

[B50] Mitton F. M. Pinedo M. L. de la Canal L. (2009). Phloem sap of tomato plants contains a DIR1 putative ortholog. *J. Plant Physiol.* 166 543–547. 10.1016/j.jplph.2008.07.002 18790546

[B51] Navarova H. Bernsdorff F. Doring A.-C. Zeier J. (2012). Pipecolic acid, an endogenous mediator of defense amplification and priming, is a critical regulator of inducible plant immunity. *Plant Cell* 24 5123–5141. 10.1105/tpc.112.103564 23221596 PMC3556979

[B52] Park S. Kaimoyo E. Kumar D. Mosher S. Klessig D. F. (2007). Methyl salicylate is a critical mobile signal for plant systemic acquired resistance. *Science* 318 113–116. 10.1126/science.1147113 17916738

[B53] Peitsch M. C. (1995). Protein modeling by E-mail. *Nat. Biotechnol.* 13 658–660. 10.1038/nbt0795-658

[B54] Perterson T. N. Brunak S. von Heijne G. Nielsen H. (2011). SignalP 4.0: discriminating signal peptides from transmembrane regions. *Nat. Methods* 8 785–786. 10.1038/nmeth.1701 21959131

[B55] Rasmussen J. B. Hammerschmidt R. Zook M. N. (1991). Systemic induction of salicylic acid accumulation in cucumber after inoculation with *Pseudmonas syringae* pv syringae. *Plant Physiol.* 97 1342–1347. 10.1104/pp.97.4.1342 16668554 PMC1081169

[B56] Rosano G. L. Ceccarelli E. A. (2014). Recombinant protein expression in *Escherichia coli*: advances and challenges. *Front. Microbiol.* 5:172 10.3389/fmicb.2014.00172 PMC4029002 24860555

[B57] Samuel D. Liu Y. Cheng C. Lyu P. (2002). Solution structure of plant non-specific lipid transfer protein-2 from rice (*Oryza sativa*). *J. Biol. Chem.* 277 35267–35273. 10.1074/jbc.M203113200 12011089

[B58] Schneider T. D. Stephens R. M. (1990). Sequence logos: a new way to display consensus sequences. *Nucleic Acids Res.* 18 6097–6100. 10.1093/nar/18.20.6097 2172928 PMC332411

[B59] Schwede T. Kopp J. Guex N. Peitsch M. (2003). SWISS-MODEL: an automated protein homology-modeling server. *Nucleic Acids Res.* 31 3381–3385. 10.1093/nar/gkg520 12824332 PMC168927

[B60] Shah J. Chaturvedi R. Chowdhury Z. Venables B. Petros R. A. (2014). Signaling by small metabolites in systemic acquired resistance. *Plant J.* 79 645–658. 10.1111/tpj.12464 24506415

[B61] Shah J. Zeier J. (2013). Long distance communication and signal amplification in systemic acquired resistance. *Front. Plant Sci.* 4:30 10.3389/fpls.2013.00030 PMC3579191 23440336

[B62] Smith-Becker J. Marois E. Huguet E. J. Midland S. L. Sims J. J. Keen N. T. (1998). Accumulation of salicylic acid and 4-hydroxybenzoic acid in phloem fluids of cucumber during systemic acquired resistance is preceded by a transient increase in phenylalanine ammonia-lyase activity in petioles and stems. *Plant Physiol.* 116 231–238. 10.1104/pp.116.1.231 9449843 PMC35162

[B63] Tamura K. Peterson D. Peterson N. Strecher G. Nai M. Kumar S. (2011). MEGA5: molecular evolutionary genetics analysis using maximum likelihood, evolutionary distance, and maximum parsimony methods. *Mol. Biol. Evol.* 28 2731–2739. 10.1093/molbev/msr121 21546353 PMC3203626

[B64] Tuzun S. Kuc J. (1985). Movement of a factor in tobacco infected with *Peronospora tabacina* adam which systemically protects against blue mold. *Physiol. Plant Pathol.* 26 321–330. 10.1016/0048-4059(85)90007-4

[B65] Vlot A. C. Liu P.-P. Cameron R. K. Park S.-W. Yang Y. Kumar D. (2008). Identification of likely orthologs of tobacco salicylic acid-binding protein 2 and their role in systemic acquired resistance in *Arabidopsis thaliana*. *Plant J.* 56 445–456. 10.1111/j.1365-313X.2008.03618.x 18643994

[B66] Vogel-Adghough D. Stahl E. Navarova H. Zeier J. (2013). Pipecolic acid enhances resistance to bacterial infection and primes salicylic acid and nicotine accumulation in tobacco. *Plant Sig. Behav.* 8 e26366 10.4161/psb.26366 PMC4091605 24025239

[B67] Whalen M. C. Innes R. W. Bent A. F. Staskawicz B. J. (1991). Identification of *Pseudomonas* syringae pathogens of *Arabidopsis* and a bacterial locus determining avirulence on both *Arabidopsis* and soybean. *Plant Cell* 3 49–59. 10.1105/tpc.3.1.49 1824334 PMC159978

[B68] Williamson M. P. (1994). The structure and function of proline-rich regions in proteins. *Biochem. J.* 297 249–260. 10.1042/bj2970249 8297327 PMC1137821

[B69] Wittek F. Hoffmann T. Kanawati B. Bichlmeier M. Knappe C. Wenig M. (2014). Arabidopsis enhanced disease susceptibility1 promotes systemic acquired resistance via azelaic acid and its precursor 9-oxo nonanoic acid. *J. Exp. Bot.* 65 5919–5931. 10.1093/jxb/eru331 25114016 PMC4203127

[B70] Xia Y. Yu K. Gao Q.-M. Wilson E. V. Navarre D. Kachroo P. (2012). Acyl CoA binding proteins are required for cuticle formation and plant responses to microbes. *Front. Plant Sci.* 3:224 10.3389/fpls.2012.00224 PMC3465942 23060893

[B71] Yeats T. H. Rose J. K. C. (2008). The biochemistry and biology of extracellular plant lipid-transfer proteins (LTPs). *Protein Sci.* 17 191–198. 10.1110/ps.073300108 18096636 PMC2222726

[B72] Yu K. Soares J. M. Mandal M. K. Wang C. Chanda B. Gifford A. N. (2013). A feedback regulatory loop between G3P and lipid transfer proteins DIR1 and AZI1 mediates azelaic-acid-induced systemic immunity. *Cell Rep.* 3 1266–1278. 10.1016/j.celrep.2013.03.030 23602565

