# Corrigendum: Orthology Analysis and *In Vivo* Complementation Studies to Elucidate the Role of DIR1 during Systemic Acquired Resistance in *Arabidopsis thaliana* and *Cucumis sativus*

**DOI:** 10.3389/fpls.2018.00460

**Published:** 2018-04-12

**Authors:** Marisa Isaacs, Philip Carella, Jennifer Faubert, Marc J. Champigny, Jocelyn K. C. Rose, Robin K. Cameron

**Affiliations:** ^1^Department of Biology, McMaster University, Hamilton, ON, Canada; ^2^Department of Molecular & Cellular Biology, University of Guelph, Guelph, ON, Canada; ^3^Plant Biology Section, School of Integrative Plant Science, Cornell University, Ithaca, NY, United States

**Keywords:** cucumber, DIR1, hydrophobic cavity, lipid transfer protein, long-distance signaling, systemic acquired resistance

An author who contributed to this paper was mistakenly left off the author list. The corrected author and affiliation lists, plus the author contribution statement are indicated below.

Marisa Isaacs^1†^, Philip Carella^1†^, Jennifer Faubert^1^, Marc J. Champigny^2^ Jocelyn K. C. Rose^3^ and Robin K. Cameron^1*^

^1^Department of Biology, McMaster University, Hamilton, ON, Canada, ^2^Department of Molecular & Cellular Biology, University of Guelph, ^3^Plant Biology Section, School of Integrative Plant Science, Cornell University, Ithaca, NY, United States.

## Author contributions

MI and PC contributed equally as first authors. Designed experiments: MI, PC, MC, JR, and RC. Performed experiments: MI, PC, MC and JF. Analyzed data: MI, PC, MC, JF, and RC. Provided reagents and equipment: JR and RC. PC and RC wrote the bulk of the manuscript, with significant contributions by MI and JR.

The original article has been updated.

### Conflict of interest statement

The authors declare that the research was conducted in the absence of any commercial or financial relationships that could be construed as a potential conflict of interest.

