# Supplementary material for: Orthology Analysis and In Vivo Complementation Studies to Elucidate the Role of DIR1 during Systemic Acquired Resistance in Arabidopsis thaliana and Cucumis sativus
Source: Front Plant Sci. 2016 May 3;7:566. doi: 10.3389/fpls.2016.00566 (PMC4854023; doi:10.3389/fpls.2016.00566)
Supplement: Supplementary file 1 [file Table_1.DOCX]

**Table S1.** Primers used in this study

| **Primer** | **Sequence (5’ - 3’)** | **Construct/Amplicon** |
| --- | --- | --- |
| BamHI-AtDIR1like-F | TCGGGATCCGGCGATTGACCTTTGTGGCATG | pET29b-DIR1like |
| XhoI-AtDIR1like-R | AGCCTCGAGACAAGTTGGGGCGTTGGTTAGG |  |
| BamHI-AtLTP2.12-F | TCGGGATCCGACTGAGGTCAAACTTTCTGGAGG | pET29b-AtLTP2.12 |
| XhoI-AtLTP2.12-R | AGCCTCGAGACAAGTAGGATAAGGAACACCAC |  |
| BamHI-CsDIR1-F | TCGGGATCCGATGGAAGTTTGCGGCGTCGACG | pET29b-CsDIR1 |
| XhoI-CsDIR1-R | GCCTCGAGAGCAGAGCAAGTGGGAGTGTTAGG |  |
| BamHI-CsDIR2-F | TCGGGATCCGCAATCCATTTGCAACATGCC | pET29b-CsDIR2 |
| XhoI-CsDIR2-R | AGCCTCGAGGCAATTTGGAGACTTAGAAATG |  |
| BamHI-DIR1^ΔCys^-F | TCGGGATCCGGCGATAGATCTCGCTGGCATG | pET29b- DIR1^ΔCys^ |
| XhoI-DIR1^ΔCys^-R | AGCCTCGAGAGCAGTTGGGGCGTTGGCTAGA |  |
| BamHI-Variants-F* | CGGGATCCGGCGATAGATCTCTGCGGCATGAGC | pET29b-Variants |
| XhoI-Variants-R* | AGCCTCGAGACAAGTTGGGGCGTTGGCTAGACC |  |
| AscI-CsDIR1-F | CACACGGGCGCGCCACCATGGAGATGGCTCAAAAGGTG | 35S:CsDIR1 (pMDC32) |
| SacI-CsDIR1-R | GGAACAGAGCTCTTAAAGGTTTAAGCAGAGCAAG |  |
| KpnI-CsDIR2-F | AGCGGTACCTTAGCAGTTGGGAGGATGAGG | 35S:CsDIR2 (pMDC32) |
| SpeI-CsDIR2-R | AGCACTAGTCTAGCAATTTGGAGACTTAGAAATG |  |
| RT-CsDIR1-F | GGTGACGGTGATGGTGGTGCTG | *CsDIR1* |
| RT-CsDIR1-R | CCAAATGAGGATAGCAACATTG |  |
| RT-CsDIR2-F | GCTATGAAAGTTGTGGCTTTAGC | *CsDIR2* |
| RT-CsDIR2-R | GCCAAAAGAAGAAAGAGCTCCCG |  |
| RT-AtDIR1-F | GATCGTGATAATGGCTATGTTGGTCGATA | *AtDIR1-EYFP* |
| RT-nEYFP-R | TCGCCGGACACGCTGAACTTGTGG |  |
| RT-EYFP-F | TGCAGTGCTTCGCCCGCTAC | *EYFP* |
| RT-EYFP-R | CGGTTCACCAGGGTGTCGCC |  |
| RT-AtACT1-F | GGCGATGAAGCTCAATCCAAACG | *ACTIN1* |
| RT-AtACT1-R | GGTCACGACCAGCAAGATCAAGACG |  |

*These primers are suitable for the amplification of the L49D, D39Q, NPH, F40Y, and AxxAxxA variants.
