# Supplementary material for: Orthology Analysis and In Vivo Complementation Studies to Elucidate the Role of DIR1 during Systemic Acquired Resistance in Arabidopsis thaliana and Cucumis sativus
Source: Front Plant Sci. 2016 May 3;7:566. doi: 10.3389/fpls.2016.00566 (PMC4854023; doi:10.3389/fpls.2016.00566)
Supplement: Supplementary file 2 [file Data_Sheet_1.DOCX]

**Supporting Document 1.** Nucleotide and amino acid sequences of DIR1 variants used in this study. Modified amino acids are highlighted.

>L49D_nt

ATGGCGAGCAAGAAAGCAGCTATGGTTATGATGGCGATGATCGTGATAATGGCTATGTTGGTCGATACATCAGTAGCGATAGATCTCTGCGGCATGAGCCAGGATGAGTTGAATGAGTGCAAACCAGCGGTTAGCAAGGAGAATCCGACGAGCCCATCACAGCCTTGCTGCACCGCTCTGCAACACGCTGATTTTGCATGTGATTGTGGTTACAAGAACTCTCCATGGCTCGGTTCTTTCGGTGTTGATCCTGAACTCGCTTCTGCTCTCCCCAAACAGTGTGGTCTAGCCAACGCCCCAACTTGTTAA

>L49D_aa

MASKKAAMVMMAMIVIMAMLVDTSVAIDLCGMSQDELNECKPAVSKENPTSPSQPCCTALQHADFAC**D**CGYKNSPWLGSFGVDPELASALPKQCGLANAPTC

>D39Q_nt

ATGGCGAGCAAGAAAGCAGCTATGGTTATGATGGCGATGATCGTGATAATGGCTATGTTGGTCGATACATCAGTAGCGATAGATCTCTGCGGCATGAGCCAGGATGAGTTGAATGAGTGCAAACCAGCGGTTAGCAAGGAGAATCCGACGAGCCCATCACAGCCTTGCTGCACCGCTCTGCAACACGCTCAATTTGCATGTCTTTGTGGTTACAAGAACTCTCCATGGCTCGGTTCTTTCGGTGTTGATCCTGAACTCGCTTCTGCTCTCCCCAAACAGTGTGGTCTAGCCAACGCCCCAACTTGTTAA

>D39Q_aa

MASKKAAMVMMAMIVIMAMLVDTSVAIDLCGMSQDELNECKPAVSKENPTSPSQPCCTALQHA**Q**FACLCGYKNSPWLGSFGVDPELASALPKQCGLANAPTC

>F40Y_nt

ATGGCGAGCAAGAAAGCAGCTATGGTTATGATGGCGATGATCGTGATAATGGCTATGTTGGTCGATACATCAGTAGCGATAGATCTCTGCGGCATGAGCCAGGATGAGTTGAATGAGTGCAAACCAGCGGTTAGCAAGGAGAATCCGACGAGCCCATCACAGCCTTGCTGCACCGCTCTGCAACACGCTGATTACGCATGTCTTTGTGGTTACAAGAACTCTCCATGGCTCGGTTCTTTCGGTGTTGATCCTGAACTCGCTTCTGCTCTCCCCAAACAGTGTGGTCTAGCCAACGCCCCAACTTGTTAA

>F40Y_aa

MASKKAAMVMMAMIVIMAMLVDTSVAIDLCGMSQDELNECKPAVSKENPTSPSQPCCTALQHAD**Y**ACLCGYKNSPWLGSFGVDPELASALPKQCGLANAPTC

>AxxAxxA_nt

ATGGCGAGCAAGAAAGCAGCTATGGTTATGATGGCGATGATCGTGATAATGGCTATGTTGGTCGATACATCAGTAGCGATAGATCTCTGCGGCATGAGCCAGGATGAGTTGAATGAGTGCAAACCAGCGGTTAGCAAGGAGAATGCTACGAGCGCTTCACAGGCTTGCTGCACCGCTCTGCAACACGCTGATTTTGCATGTCTTTGTGGTTACAAGAACTCTCCATGGCTCGGTTCTTTCGGTGTTGATCCTGAACTCGCTTCTGCTCTCCCCAAACAGTGTGGTCTAGCCAACGCCCCAACTTGTTAA

>AxxAxxA_aa

MASKKAAMVMMAMIVIMAMLVDTSVAIDLCGMSQDELNECKPAVSKEN**A**TS**A**SQ**A**CCTALQHADFACLCGYKNSPWLGSFGVDPELASALPKQCGLANAPTC

>NPH_nt

ATGGCGAGCAAGAAAGCAGCTATGGTTATGATGGCGATGATCGTGATAATGGCTATGTTGGTCGATACATCAGTAGCGATAGATCTCTGCGGCATGAGCGCTGATGAGTTGGCTGAGTGCGCTCCAGCGGTTAGCAAGGAGAATCCGACGAGCCCATCACAGCCTTGCTGCACCGCTCTGCAACACGCTGATTTTGCATGTCTTTGTGGTTACAAGAACTCTCCATGGCTCGGTTCTTTCGGTGTTGATCCTGAACTCGCTTCTGCTCTCCCCAAACAGTGTGGTCTAGCCAACGCCCCAACTTGTTAA

>NPH_aa

MASKKAAMVMMAMIVIMAMLVDTSVAIDLCGMS**A**DEL**A**EC**A**PAVSKENPTSPSQPCCTALQHADFACLCGYKNSPWLGSFGVDPELASALPKQCGLANAPTC

>DIR1^ΔCys^_nt

ATGGCGAGCAAGAAAGCAGCTATGGTTATGATGGCGATGATCGTGATAATGGCTATGTTGGTCGATACATCAGTAGCGATAGATCTCGCTGGCATGAGCCAGGATGAGTTGAATGAGGCTAAACCAGCGGTTAGCAAGGAGAATCCGACGAGCCCATCACAGCCTGCTGCTACCGCTCTGCAACACGCTGATTTTGCAGCTCTTGCTGGTTACAAGAACTCTCCATGGCTCGGTTCTTTCGGTGTTGATCCTGAACTCGCTTCTGCTCTCCCCAAACAGGCTGGTCTAGCCAACGCCCCAACTGCTTAA

> DIR1^ΔCys^_aa

MASKKAAMVMMAMIVIMAMLVDTSVAIDL**A**GMSQDELNE**A**KPAVSKENPTSPSQP**AA**TALQHADFA**A**L**A**GYKNSPWLGSFGVDPELASALPKQ**A**GLANAPT**A**
