# Supplementary figures and images for: Orthology Analysis and In Vivo Complementation Studies to Elucidate the Role of DIR1 during Systemic Acquired Resistance in Arabidopsis thaliana and Cucumis sativus
Source: Front Plant Sci. 2016 May 3;7:566. doi: 10.3389/fpls.2016.00566 (PMC4854023; doi:10.3389/fpls.2016.00566)

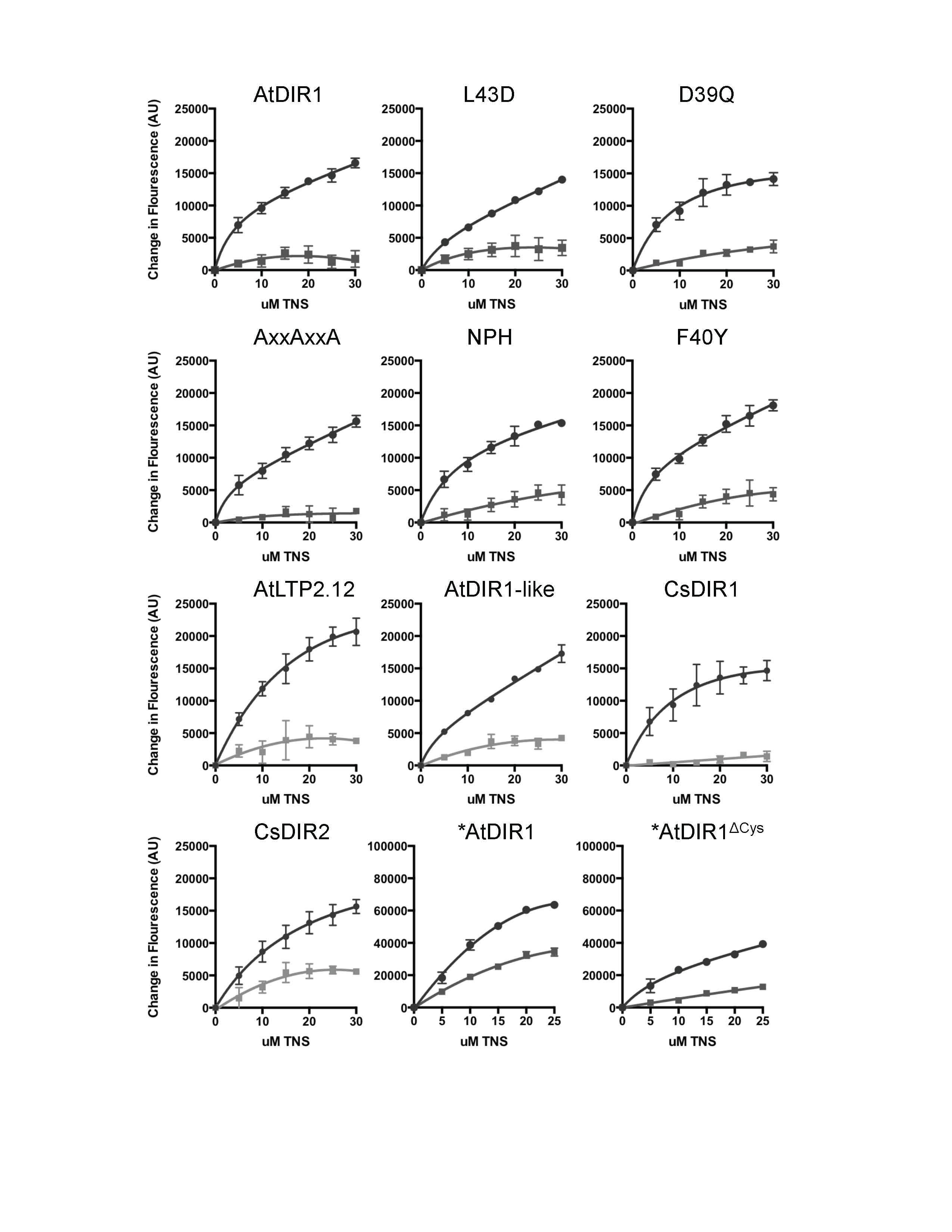

Supplement: FIGURE S1 — Individual in vitro TNS (6,P-toluidinylnaphthalene-2-sulfonate) binding assays of recombinant AtDIR1, AtDIR1-like, AtLTP2.12, CsDIR1, CsDIR2, and DIR1 variant proteins. Natured protein (black lines, circles) is compared to denatured protein for sample (gray lines, squares). Increasing concentrations of TNS were added to each Rosetta Gami E. coli purified protein lacking the ER signal sequence. TNS binding curves were generated in PRISM6 by non-linear curve fitting for one site saturation binding. Proteins were denatured by boiling in 6 M Urea. Samples were excited at 320 nm and emission at 437 nm and the change in fluorescence was calculated for three replicates. Error bars represent the standard deviation. An asterisk (∗) indicates proteins that were analyzed using a TECAN M1000 rather than the Gen5 BioTek fluorometer. [file Image_1.JPEG]
