# Supplementary material for: Orthology Analysis and In Vivo Complementation Studies to Elucidate the Role of DIR1 during Systemic Acquired Resistance in Arabidopsis thaliana and Cucumis sativus
Source: Front Plant Sci. 2016 May 3;7:566. doi: 10.3389/fpls.2016.00566 (PMC4854023; doi:10.3389/fpls.2016.00566)
Supplement: FIGURE S2 — Batch Reproducibility of the TNS binding assay. TNS binding curves of identical proteins purified from different batches was performed for AtDIR1 (A) and AtDIR1- like (B). Student’s t-tests (p < 0.05) of natured proteins from batch 1 and batch 2 did not identify statistically significant differences in TNS binding capacity (means of denatured proteins were not compared). Values represent the mean ± standard deviation of 3 technical replicates. These assays were performed using a Gen5 Synergy 4 (BioTek) plate reader. [file Image_2.PDF]

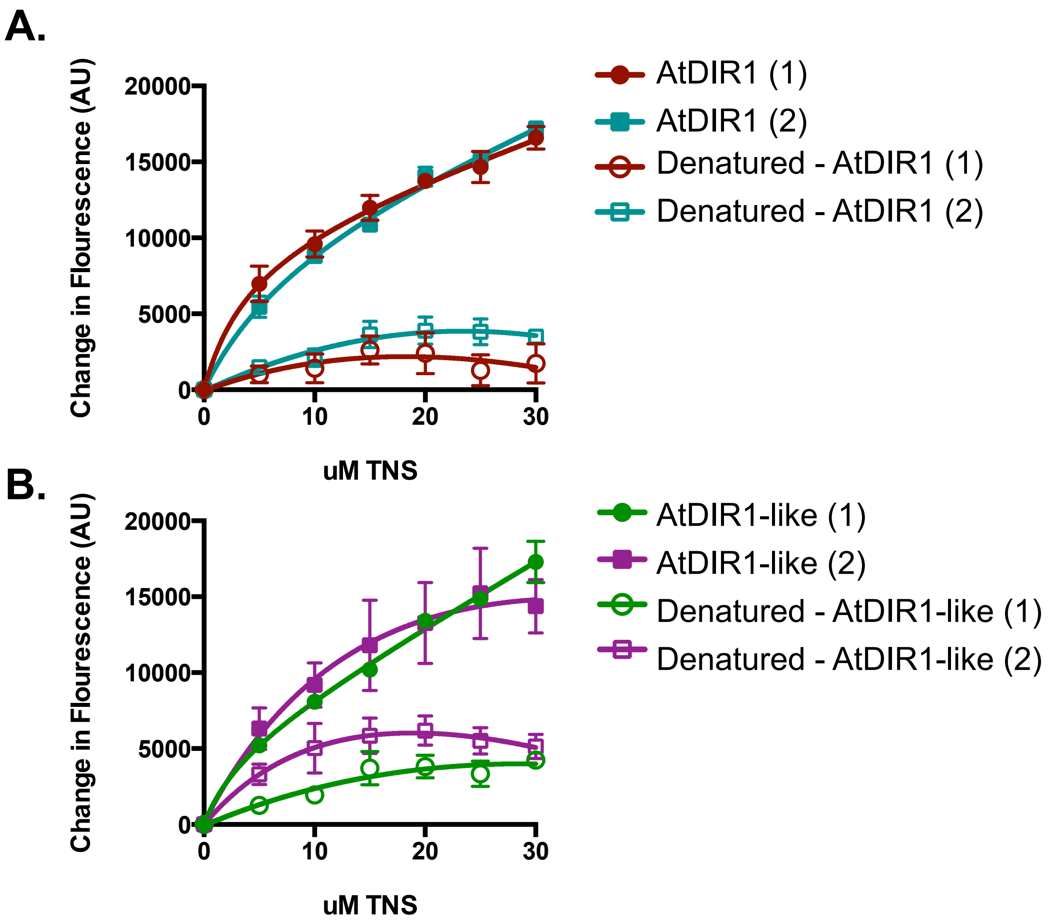

Fig. S2 – Batch Reproducibility of the TNS binding assay. TNS binding curves of identical proteins purified from different batches was performed for AtDIR1 (A) and AtDIR1-like (B). Student's t-tests ($p < 0.05$) of natured proteins from batch 1 and batch 2 did not identify statistically significant differences in TNS binding capacity (means of denatured proteins were not compared). Values represent the mean +/- standard deviation of 3 technical replicates. These assays were performed using a Gen5 Synergy 4 (BioTek) plate reader.
