# Supplementary material for: Orthology Analysis and In Vivo Complementation Studies to Elucidate the Role of DIR1 during Systemic Acquired Resistance in Arabidopsis thaliana and Cucumis sativus
Source: Front Plant Sci. 2016 May 3;7:566. doi: 10.3389/fpls.2016.00566 (PMC4854023; doi:10.3389/fpls.2016.00566)
Supplement: FIGURE S3 — Confirmation of successful Agrobacterium-mediated expression in dir1-1. RT-PCR analysis was used to monitor the expression of EYFP, AtDIR1-EYFP, CsDIR1, and CsDIR2 four days post agro-infiltration of dir1-1 leaves (3.5 week-old plants). Expression of the endogenous ACTIN1 housekeeping gene was monitored as a loading control. Primers for RT-PCR analysis can be found in Table S1. This was performed twice with similar results. [file Image_3.PDF]

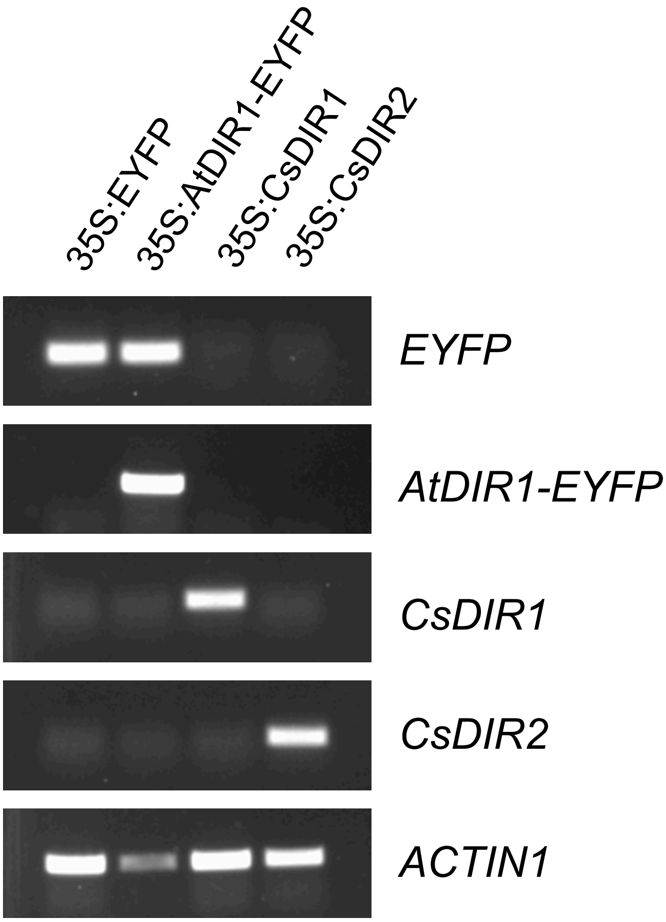

Figure S3 Confirmation of successful Agrobacterium-mediated expression in *dir1-1*. RT-PCR analysis was used to monitor the expression of *EYFP*, *AtDIR1-EYFP*, *CsDIR1*, and *CsDIR2* four days post agro-infiltration of *dir1-1* leaves (3.5 week-old plants). Expression of the endogenous *ACTIN1* housekeeping gene was monitored as a loading control. Primers for RT-PCR analysis can be found in Table S1. This was performed twice with similar results.
